# Phase-separation facilitated one-step fabrication of multiscale heterogeneous two-aqueous-phase gel

**DOI:** 10.1038/s41467-023-38394-9

**Published:** 2023-05-16

**Authors:** Feipeng Chen, Xiufeng Li, Yafeng Yu, Qingchuan Li, Haisong Lin, Lizhi Xu, Ho Cheung Shum

**Affiliations:** 1 Department of Mechanical Engineering, The University of Hong Kong, Pokfulam Road, Hong Kong (SAR), China; 2 Advanced Biomedical Instrumentation Centre, Hong Kong Science Park, Shatin, New Territories, Hong Kong (SAR), China

**Keywords:** Gels and hydrogels, Gels and hydrogels, Polymers

## Abstract

Engineering heterogeneous hydrogels with distinct phases at various lengths, which resemble biological tissues with high complexity, remains challenging by existing fabricating techniques that require complicated procedures and are often only applicable at bulk scales. Here, inspired by ubiquitous phase separation phenomena in biology, we present a one-step fabrication method based on aqueous phase separation to construct two-aqueous-phase gels that comprise multiple phases with distinct physicochemical properties. The gels fabricated by this approach exhibit enhanced interfacial mechanics compared with their counterparts obtained from conventional layer-by-layer methods. Moreover, two-aqueous-phase gels with programmable structures and tunable physicochemical properties can be conveniently constructed by adjusting the polymer constituents, gelation conditions, and combining different fabrication techniques, such as 3D-printing. The versatility of our approach is demonstrated by mimicking the key features of several biological architectures at different lengths: macroscale muscle-tendon connections; mesoscale cell patterning; microscale molecular compartmentalization. The present work advances the fabrication approach for designing heterogeneous multifunctional materials for various technological and biomedical applications.

## Abstract (web summary)

Preparing heterogeneous hydrogels with distinct phases is desirable for the mimicking of biological tissues, but generally requires complex techniques. Here, the authors report a method that uses phase separation to fabricate two-aqueous phase soft materials with distinct properties at different length scales.

## Introduction

**Fig. 1:**
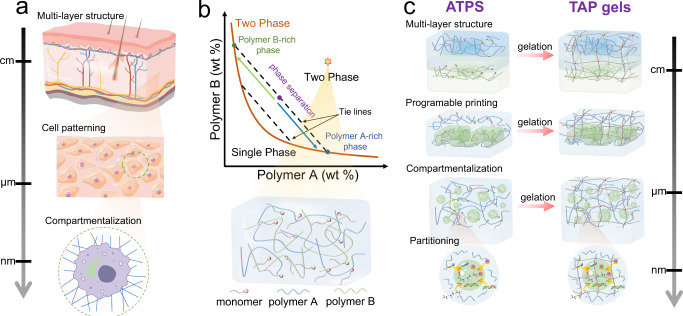
TAP gels mimicking hierarchical heterogeneous biological tissues. **a** Schematic illustration of biological tissues with heterogeneous structures at different length scales: human skins with distinct layers in parallel at macroscales; cell patterns within each tissue layer at mesoscales; cellular compartmentalization at microscales. **b** Schematic phase diagram of an aqueous two-phase system (ATPS) system consisting of polymer A and polymer B. A bimodal curve colored in deep orange is delineated as the critical boundary between the single-phase regime and the two-phase regime. An initial point in the two-phase regime phase separates into two thermal equilibrium aqueous phases, the polymer A-rich phase and polymer B-rich phase, following a tie line. **c** Schematic figures of multiscale two-aqueous-phase (TAP) gels as a resemblance of biological tissues, including multi-layer structures at macroscales, programable printed structures at mesoscales, and molecular compartmentalization and partitioning at microscales.

Biological tissues are hierarchically constructed with heterogeneous layers or structures in a wide range of length scales^1–3^. The heterogeneity encodes diverse physicochemical properties of biological tissues, such as stiffness, stretchability, porosity, and preferential partitioning of molecules^3,4^. Importantly, heterogeneous biological tissues spatially organize different types of cells and synergistically operate them to perform the physiological functions of living organisms^3^. For example, human skins are comprised of several layers with their compositions varying in cell types, proteins, and networks, which possess distinct thickness, stiffness, and physiological functions in protecting the human body from external invasions of fungi or bacteria and sensing mechanical stimuli^1^ (Fig. 1a). Another example at smaller scales is that cells precisely compartmentalize biomacromolecules into membraneless condensates as an alternative way to achieve spatiotemporal organization of intracellular environments and regulate many physiological functions in addition to the common scenario of lipid bilayer-enclosed organelles^5,6^. Mimicking multiscale biological tissues at different length scales not only produces model systems that may facilitate our understanding of how nature constructs sophisticated living architectures but also forms the basis for designing multifunctional soft materials from an engineering perspective. These tissue-like materials could be beneficial for a variety of studies and applications, such as investigating the cell dynamics and migrations in tissue-like environments, designing biocompatible bridging materials for bio-interfaces with monitoring devices, and fabricating healthcare materials for tissue healing and regeneration^7–9^.

Hydrogel, a crosslinked polymer network infiltrated with a high content of water, has emerged as a promising material for imitating processes, behaviors, and architectures observed in biological tissues and has been deployed for plenty of biomedical applications^10–13^. For example, with substantial efforts devoted in past decades, physicochemical properties of homogeneous hydrogels, such as toughness^14–17^, self-healing^18–20^, fatigue resistance^21,22^, self-growth^23^, and adhesiveness^24,25^, have been extensively studied and improved to capture biology-defining features, benefiting many disciplines, including tissue engineering, wound dressing, drug release, and soft robots^13,23,24,26–28^. Recently, various studies have reasoned the importance of hydrogels’ heterogeneity in fabricating multifunctional soft materials^10,29–32^, such as constructing multifunctional stimuli-controlled soft robots^33^, promoting tissue regeneration^34,35^, and compartmentalizing incompatible enzymatic reactions^36^. These heterogeneous hydrogels are usually fabricated by traditional approaches, including patterning^33,37,38^, 3D printing^35,39,40^, and microfluidics^36,41,42^, combined with a conventional layer-by-layer methodology which sequentially integrates different hydrogel domains. However, these approaches are tedious due to the subsequent surface modification and connection of interfaces to build heterogeneous hydrogels; the resulting structures may also suffer weak interfacial mechanics between post-connected gels^43,44^. Moreover, conventional approaches are usually restricted to fabricating simple geometries (e.g., mostly on flat surfaces), which remain difficult to fully imitate biological tissues with complicated architectures, diverse physicochemical properties, and hierarchical length scales^10^. In addition, the layer-by-layer approach for designing heterogeneous hydrogels becomes inapplicable at small length scales such as at micro-/nanometers. Therefore, a facile, versatile, and controllable approach, which can conveniently construct heterogeneous hydrogels at different length scales imitating diverse structures and functions of biological tissues, is highly desired.

Nature shows us a facile way to build living organisms based on molecular interactions among different building blocks^45^. For example, recent advances in cell biology, especially the role of membraneless organelles, suggest that intracellular environments can be spatially organized by biomolecular condensates through the aqueous phase separation driven by multivalent interactions among proteins and RNAs^5,46^. As a simple format of phase separation, an aqueous two-phase system (ATPS) triggered by segregative interactions between incompatible constituent polymers has been considered a simple model system to study biological questions^47,48^ as well as deployed in many industrial applications, such as extraction, separation, and purification of biomolecules and cell organelles^49,50^. Such phase separation phenomenon creates immiscible aqueous phases with distinct properties, such as viscosity and surface tension, depending on polymer compositions and environmental conditions^51^. In previous studies, integration of ATPS with 3D bioprinting has been demonstrated to construct cell patterns and vascularized architectures with selective partitioning of molecules, which facilitates compartmentalized chemical reactions^52–56^. This ATPS-based bioprinting approach exhibits unique performance and advantages, such as high reagent activity and cell viability due to the fully aqueous environment, suppressed collapse of printed liquid structures accounting for the low interfacial tension, bottom-up construction of ultrasoft soft robots^53–56^. Nevertheless, the structures constructed by interfacial polymer complexation have an interior aqueous environment and thus are very soft and fragile with the stiffness of only hundreds of pascals^56^, which limits their applications. In contrast, biological tissues are usually supported by polymer networks with remarkable mechanical properties. To overcome the challenge, a gelation process that crosslinks monomers into polymer networks may offer greater and more controllable mechanical stability for multiscale structures that are pre-constructed within ATPS.

The present work explores a facile and versatile method to fabricate heterogeneous hydrogels at different length scales. Our method enables the one-step fabrication of heterogeneous hydrogels by introducing immiscible aqueous domains via phase separation prior to crosslinking pre-dissolved monomers, termed two-aqueous-phase (TAP) gels (Fig. 1c). The heterogeneous hydrogels obtained from our one-step fabrication method exhibit enhanced interfacial mechanics, compared to their counterparts fabricated by conventional layer-by-layer methods. For example, the critical strain and fracture energy is improved two and four times, respectively, for a TAP gel composed of polyethylene glycol (PEG), dextran (DEX), and polyacrylamide (PAM). Furthermore, we show that the physicochemical properties, including stiffness, stretchability, and molecular partitioning, and the structures of TAP gels can be readily tuned by controlling polymer compositions and gelation conditions and combining different fabrication techniques. Consequently, TAP gels are constructed to imitate features of several representative biological architectures at different length scales. At a macroscale, the connection between soft muscles and stiff tendons is imitated by a TAP gel containing different gel phases with distinct mechanical properties. At a mesoscale, embedded cell patterns are constructed in a programmable manner using 3D printing, mimicking biological tissues that consist of different types of cells. At a microscale close to cell dimensions, a structure of micro-gels embedded in a bulk hydrogel is designed, with control over the size distribution of micro-gels, the partition, and the release of molecules. This property may benefit the designing of drug delivery systems that can selectively load drugs at high concentrations and further release them at specific locations. Therefore, this study represents a significant advancement in the fabrication approaches for constructing heterogeneous multifunctional materials for a wide range of technological and biomedical applications.

## Results

### Mechanism and phase diagram

Similar to biological tissues, physicochemical diversity due to the different physical nature of polymers, salts, or surfactants in a mixture allows subset components to establish preferential interactions with one another, leading to segregative phase separation into multiple phases in aqueous environments^50^. Multiple phases can be hierarchically organized based on their physical properties, such as interfacial tension, hydrophilicity, and density. ATPS is a well-studied phase separation phenomenon, which is mainly driven by repulsive interactions among incompatible polymers/salts/surfactants^49^. A classic example of ATPS is the mixture of PEG and DEX, where the system separates into two immiscible aqueous phases at equilibrium. One phase is enriched with PEG, and the other is enriched with DEX^49^. The physicochemical diversity inherent in ATPS arises from the fact that different aqueous phases have distinct physical and chemical properties, such as viscosity, hydrophobicity, as well as affinity for various molecules. The spontaneous generation of immiscible aqueous phases and physicochemical diversity make ATPS an attractive platform for fabricating heterogeneous hydrogels at different length scales by crosslinking the pre-dissolved monomers into polymer networks (Fig. 1c).

Phase separation is a thermodynamically favorable process where entropic contribution favoring mixing is smaller than enthalpic contribution caused by incompatible interactions among the polymers^49^. A phase diagram is commonly used to guide the preparation of multiphase systems. The phase diagram can be acquired in both experimental and theoretical approaches to delineate the regime where phase separation will occur as a function of two polymers’ concentrations (Fig. 1b). When the concentrations of polymers are above critical concentrations, the mixture will phase-separate into two immiscible phases following a tie line: polymer-A rich and polymer-B rich phases (Fig. 1b). At an equilibrium state, two phases have equal chemical potentials as well as osmotic pressures^57^. All equilibrium points constitute a binodal curve that separates the single-phase regime from the two-phase regime. Binodal curves can be affected by many parameters, such as polymer length, pH, and temperature^58^. Among the others, polymer length (or molecular weight) is one of the commonly used parameters to control binodal curves in the phase diagram. For example, we have measured phase diagrams for three groups of ATPS consisting of PEG (Mw ~8000) and dextran with different molecular weights (Mw ~10,000, 40,000, and 500,000) (Supplementary Fig. 4). The binodal curves gradually shift to the bottom left corner, indicating that the phase separation can happen at lower concentrations when using dextran with higher molecular weights (Supplementary Fig. 5). Above experimental results are consistent with the theoretical analysis by using a mean-field theory, for example, the Flory–Huggins model, which quantifies the entropic and enthalpic contributions (Supplementary Note 1). With the phase diagrams and a basic understanding of phase separation, ATPS can be prepared for the further design of heterogeneous hydrogels.

### Heterogeneous TAP gel at the macroscale

**Fig. 2:**
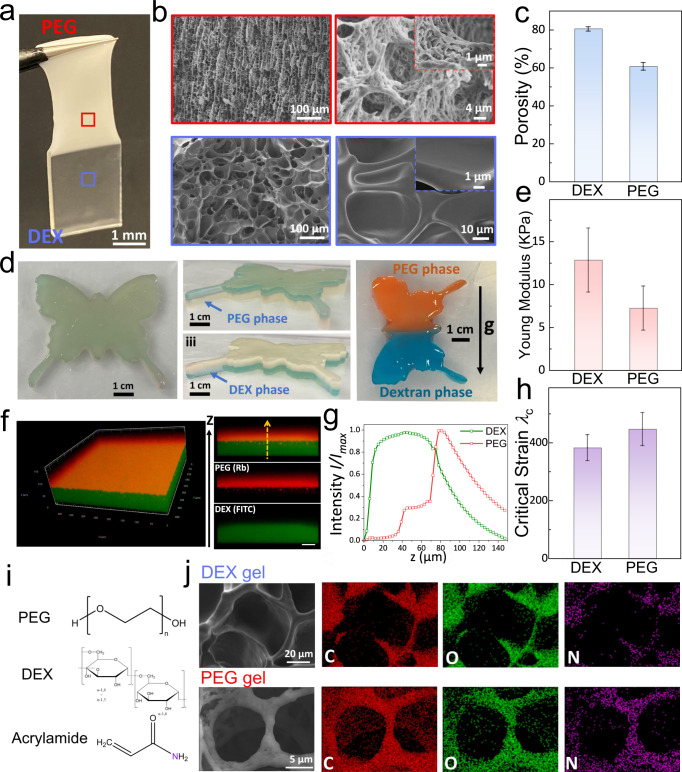
Macroscale TAP gels with heterogeneous properties. **a** Photograph of a TAP gel consisting of a PEG gel phase at the top and a DEX gel phase at the bottom. **b** SEM images of microscale structures of a PEG gel (red square) and a DEX gel (light blue square) showing their distinct porous networks. **c** Porosity of the PEG gel (${\phi }_{{{\rm{PEG}}}}=60.8\pm 2.0$) and the DEX gel (${\phi }_{{{\rm{DEX}}}}=80.6\pm 1.2$) calculated based on acquired SEM images. Error bars indicate mean ± SD (*n* = 3 independent samples). **d** A butterfly TAP gel with different arrangements of two gel phases relative to gravity. **e** Young Modulus $E$ of the PEG gel (${E}_{{{\rm{PEG}}}}=7.3\pm 2.6$ kPa) and DEX gel (${E}_{{{\rm{DEX}}}}=12.9\pm 3.7$ kPa) as the slope of stress–strain curves. Error bars indicate mean ± SD (*n* = 3 independent samples). **f** Confocal microscopy images of a TAP gel showing the phase transition between two phases where the DEX gel is labeled with fluorescein isothiocyanate (FITC) and the PEG gel is labeled with Rhodamine (Rh) (scale bar is 50 µm). **g** Relative fluorescence intensity *I*/*I*_max_ profile of FITC-DEX and Rh-PEG across the interface. *I*_max_ represents the maximum value of fluorescence intensity in the absorption unit (a.u.). **h** Critical strain ${\lambda }_{c}$ of the PEG gel (${\lambda }_{C}=447\pm 57.1\%$) and DEX gel (${\lambda }_{C}=383\pm 45.0\%$) as measured by the maximum strain before fracture. Error bars indicate mean ± SD (*n* = 3 independent samples). **i** Chemical structures of PEG, DEX, and acrylamide, respectively. Only acrylamide contains the nitrogen element marked in purple. **j** Energy dispersive X-ray (EDX) images of the DEX gel and PEG gel demonstrating the successful formation of the PAM hydrogel network.

To resemble macroscopic skin-like structures, a multi-layer TAP gel is constructed. A turbid mixture consisting of PEG 8 K and DEX T40 with concentrations in the two-phase regime is first prepared (Supplementary Fig. 6a). After reaching equilibrium, the mixture separates into two transparent bulk phases, where the PEG phase sits on top of the DEX phase due to their difference in density (Supplementary Fig. 6b). Next, by crosslinking the pre-dissolved monomers (acrylamide, 10 wt%) with crosslinkers (N, N’-methylenebisacrylamide, MBAA) at 0.5% weight ratio of monomers under UV light for 15 min, TAP gels with two distinct gel phases can be formed (Fig. 2a). PAM hydrogel networks are successfully formed in both gel phases, as confirmed by an Energy dispersive X-ray (EDX) analysis where the nitrogen element from acrylamide has been detected in both phases (Fig. 2i, j). In addition, the EDX analysis reveals that the amount of Nitrogen (N) element is much lower than carbon (C) and oxygen (O) (Supplementary Fig. 7), consistent with chemical structures of PEG, DEX, and acrylamide (Fig. 2i). The interface between two gels is further characterized by a Confocal laser scanning microscopy (CLSM) using fluorescein isothiocyanate-labeled DEX (FITC-DEX) and Rhodamine-labeled PEG (Rh-PEG) (Fig. 2f). A microscale phase transition from the DEX-rich phase at the bottom to PEG-rich phase at the top is observed, as shown in Fig. 2g. High-magnification CLSM images at the interface suggest there is a certain amount of PEG polymer left in DEX-rich phase and a certain amount of DEX polymer in PEG-rich phase (Supplementary Fig. 8). The exact polymer compositions of each phase can be determined through a lever rule based on the experimentally obtained phase diagram (Supplementary Note 2). Results show that the PEG-rich phase contains 2.6 wt% DEX and 14 wt% PEG; in contrast, DEX-rich phase comprises 24.8 wt% DEX and 2.2 wt% PEG (Supplementary Fig. 3).

More complicated morphologies of TAP gels can be constructed using different fabrication molds. For example, a butterfly TAP gel can be constructed using a pre-designed mold, and the arrangement of different gel layers can be readily tuned (Fig. 2d). In addition, by combining the phase separation of a solution comprised of three incompatible polymers (PEG, DEX, and Poly(2-ethyl-2-oxazoline) (PEtOx)), a heterogeneous hydrogel with three different gel phases is fabricated (Supplementary Fig. 9). Following the same concept, a heterogeneous hydrogel with more layers can be constructed in principle, which is extended as multiple aqueous phases (MAP) gels^50^. Heterogeneous MAP gels with multiple layers induced by phase separation are reminiscent of multi-layer structures in human skin (Fig. 1a). In this study, the TAP gel with two gel phases will be the focus as a start.

Two gel phases of the TAP gel are further characterized to show heterogeneous physical properties in terms of transparency and mechanical strength. Macroscopically, the PEG gel phase is more opaque and manifests as a milky white color, whereas the DEX gel phase is more transparent (Fig. 2a). Having differences in transparency may imply that a post-phase separation occurs in the PEG-rich during the polymerization and crosslinking of acrylamide monomers^59^. We observed that only the PEG-rich phase is transformed from a transparent solution (Supplementary Fig. 6b) to an opaque hydrogel after the crosslinking process. This observation is consistent with the previous study that shows PEG can phase separate from PAM, while DEX cannot^50^. In the present work, the post-phase separation may generate poly-dispersed nano/microscale emulsion droplets, which scatter more light and result in the opacity of the gels after crosslinking^59–61^. This post-phase separation remains to be confirmed, for instance, by small-angle X-ray/neutron scattering (SAXS/SANS) techniques^62^ in future studies. Secondly, the opaque PEG gel may be attributed to the difference in its microscale architectures compared to the DEX gel. While interpenetrated polymer networks are observed in both gel phases, the PEG gel phase has denser and smaller pores formed by entangled, bundled micro/nano-scale polymer fibers compared with the DEX gel phase as revealed by scanning electron microscopy (SEM) (Fig. 2b). Consequently, the porosity ($\phi$) of two gel phases differs as the ratio of void areas to the whole area, showing that the PEG gel phase has smaller porosity (${\phi }_{{{\rm{PEG}}}}=60.8\pm 2.0$) than the DEX gel phase (${\phi }_{{{\rm{DEX}}}}=80.6\pm 1.2$) (Fig. 2c). This difference in porosity between PEG gel and DEX gel at microscales may contribute to their distinct macroscopic transparency.

In addition, two phases of the TAP gel have heterogeneous mechanical strength characterized by Young Modulus ($E$) as a measure of stiffness and critical strain (${\lambda }_{C}$) representing the maximum strain before fracture. Results show that the DEX gel is stiffer (${E}_{{{\rm{DEX}}}}=12.9\pm 3.7$ kPa) than the PEG gel (${E}_{{{\rm{PEG}}}}=7.3\pm 2.6$ kPa), whereas it is less stretchable (${\lambda }_{C}=383\pm 45.0\%$) than the PEG gel (${\lambda }_{C}=447\pm 57.1\%$), as obtained from a tensile tester (Fig. 2e, Fig. 2h, and Supplementary Fig. 10). Differences in stiffness and stretchability between PEG and DEX gels may be caused by biased polymer constituents, such as the selective partition of monomers between two phases, thus forming distinct polymer networks at the microscale, all of which could lead to different mechanical properties^62,63^. The stiffness and critical strain of each phase in the TAP gel can be tuned by varying the crosslinker concentration (Supplementary Fig. 11a) in line with previous studies^14^. Remarkably, we can achieve highly stretchable DEX hydrogel with ${\lambda }_{C}$ up to around 2900% at a low crosslinker concentration (0.06 wt% of monomers) (Supplementary Fig. 11b). This demonstrates that we can readily modulate the physical properties of TAP gel by controlling gelation conditions, such as the crosslinker concentration.

### TAP gels with contrast stiffness mimicking the muscle-tendon connection

**Fig. 3:**
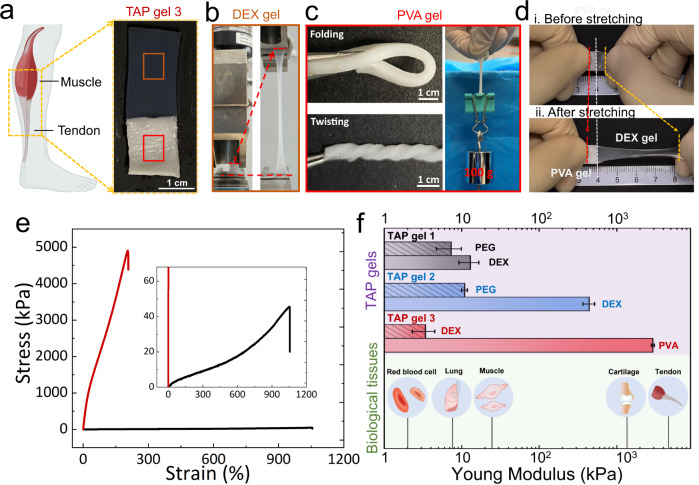
TAP gels with contrast stiffness to mimic the muscle-tendon connection. **a** TAP gel 3 consisting of the PVA gel and DEX gel is constructed to mimic the muscle–tendon connection. Photographs show that **b** the DEX gel is soft and stretchable, but **c** the PVA gel is tough and stiff to withstand a heavy load. **d** PVA and DEX gels exhibit different strains under the same strain before and after stretching in the TAP gel 3, demonstrating their distinct mechanical strengths. **e** A representative stress–strain curves of the PVA (red) and DEX gel (black) measured by a tensile tester. Based on obtained stress–strain curves, the slopes represent the Young modulus of gels, $E$; the maximum strain that hydrogels can sustain before fracture is the critical strain, ${\lambda }_{C}$. **f** TAP gels show the contrast between moduli in a wide range to mimic biological tissues. TAP gel 1: ${E}_{{{\rm{DEX}}}}=12.9\pm 3.7\,{{{\rm{kPa}}}}$ and ${E}_{{{\rm{PEG}}}}=7.3\pm 2.6\,{{{\rm{kPa}}}}$; TAP gel 2: ${E}_{{{\rm{DEX}}}}=441.7\pm 76.4\,{{{\rm{kPa}}}}$ and ${E}_{{{\rm{PEG}}}}=10.9\pm 0.9\,{{{\rm{kPa}}}}$; TAP gel 3: ${E}_{{{\rm{DEX}}}}=3.4\pm 1.2\,{{{\rm{kPa}}}}$ and ${E}_{{{\rm{PVA}}}}=3.0\pm 0.1\,{{{\rm{MPa}}}}$; Error bars indicate mean ± SD (*n* = 3 independent samples). The muscle-tendon cartoons in (**a**) and (**f**) were created with BioRender.com.

Biological tissues exhibit heterogeneous mechanical properties with moduli ranging from tens of kPa, such as lung and muscle, to a few MPa, including tendons and cartilages (Fig. 3f)^64^. Most importantly, tissues with contrasting mechanical properties are well connected to perform specific functions in the human body. For example, the tendon serves as a resilient connective tissue between muscles and bones to transmit the force exerted by muscles to bones, and it plays a critical role in a plethora of human body movements. However, our current TAP gels are too soft (small $E$) to withstand sufficient load for practical applications, and it cannot exhibit a wide range of moduli typically observed in biological tissues, such as artificial tendons. Moreover, the ratio of moduli between two gels, denoted as $\kappa \,( > 1)$, is insufficient to mimic the muscle-tendon connection. Meanwhile, it is observed that modulating ATPS compositions, such as increasing the molecular weight or changing polymer species, has negligible effects on enhancing the mechanical properties of TAP gels (Supplementary Fig. 12), although they are previously used in shifting phase diagram of ATPS (Supplementary Fig. 4) and altering interfacial tensions^51^.

To further improve the mechanical strength of TAP gels, we strategically change constituent polymers and gelation conditions. To make it clear for comparison, the ATPS consists of PEG 8 K and DEX T40 formed in the above section and is named TAP gel 1. In literature, double network (DN) hydrogels have been demonstrated as promising soft materials with good mechanical properties due to complementary interpenetrating networks and multiple energy dissipation mechanisms^65^. Therefore, we add sodium alginate (10 wt%) into TAP gel 1 and sink it into calcium chloride solutions to form a DN hydrogel named TAP gel 2. Sodium alginate forms a second ionically crosslinked network in the presence of a crosslinker (calcium chloride), which coexists with the first covalent polyacrylamide network^14,66^. With this modification, the modulus of DEX gel is significantly improved from $12.9\pm 3.7\,{{{\rm{kPa}}}}$ (TAP gel 1) to $441.7\pm 76.4\,{{{\rm{kPa}}}}$ (TAP gel 2), as shown in Supplementary Fig. 13. The observed enhancement resonates that the formation of sodium alginate network can efficiently dissipate energy, thereby increasing the modulus and stiffness of hydrogels^14,16^. However, the modulus of PEG gel has not been significantly increased ($7.3\pm 2.6\,{{{\rm{kPa}}}}$ in TAP gel 1 and$\,10.9\pm 0.9\,{{{\rm{kPa}}}}$ in TAP gel 2), which indicates that the alginate network may not be formed in PEG gel. This is explained that most of the sodium alginate will partition into the DEX aqueous phase during the phase separation rather than PEG aqueous phase due to its higher affinity with DEX^49,67^. Such preferential partitioning allows for the fine-tuning of the mechanical properties of one gel while leaving the other unchanged. Consequently, it increases the contrast between the mechanical properties of two connected gels. For example, the contrast between the moduli of two gels increases from 1.8-fold in TAP gel 1 to 40.4-fold in TAP gel 2. However, despite being improved, TAP gel 2 with the addition of sodium alginate does not yet exhibit a comparable mechanical strength to that of biological tendons that typically exhibit a modulus in the MPa scale.

Next, we select an alternative ATPS system consisting of polyvinyl alcohol (PVA) and DEX to form TAP gel 3. PVA hydrogel has been previously demonstrated as a candidate material for mimicking tendons with hierarchical structures by combining the directional freeze-casting and salting-out process^15^. In TAP gel 3, the polyacrylamide network is first formed by crosslinking acrylamide monomers (10 wt%) with MBAA crosslinkers (0.5 wt% of monomers) under UV light for 15 min. Then the TAP gel 3 is immersed into a salt bath containing 1.5 M sodium citrate for 4 days. Consequently, PVA hydrogel becomes very stiff with a mechanical modulus ${E}_{{{\rm{PVA}}}}$ up to $3.0\pm 0.1\,{{{\rm{MPa}}}}$ as characterized by a tensile tenser (Fig. 3e). This significant improvement in mechanical properties of PVA hydrogel has been attributed to the efficient energy dissipation and structural densification due to the formation of hydrogen bonds and crystalline domain^15^, which are formed during the salting-out process. Moreover, experimental results demonstrate that the PVA gel, without undergoing the salting-out process, exhibits a significantly low modulus of ~44.7 kPa (Supplementary Fig. 14). TAP gel 3 can withstand folding, twisting, and lift a weight that is ~200 times heavier than its own weight (Fig. 3c). At the same time, the DEX gel remains very soft (${E}_{{{\rm{DEX}}}}=3.4\pm 1.2$ kPa) and stretchable with ${\lambda }_{C}=898.7\pm 109\%$ (Fig. 3b, e). TAP gel 3 shows a higher contrast between the modulus of the two phases with the ratio $\kappa=869\,({E}_{{{\rm{PVA}}}}/{E}_{{{\rm{DEX}}}}).$ As a result, TAP gel 3 can successfully mimic the mechanical characteristics of the muscle-tendon connection. As another demonstration, the DEX gel phase can be stretched around 8 times longer than its original length, but the PVA gel phase is rarely stretchable (Fig. 3d). In addition to biomimetic applications, the combination of very soft and very stiff hydrogels has the potential to serve as the bridging interface between soft human tissues and rigid electric devices^9^.

We argue that by strategically controlling constituent polymers and gelation conditions, the mechanical properties of TAP gels can be further fine-tuned to meet different requirements for various applications. In particular, many established approaches for improving the mechanical properties of homogeneous hydrogels can potentially be applied to develop TAP gels^12^, as there is no intrinsic conflict between ATPS and fabrication procedures. For example, we have proved that the formation of a second physical network can significantly enhance the mechanical strength of the whole TAP gel, which has been widely described in literature^14,15^. Our primary concept is that ATPS serves as a versatile integration platform creating immiscible aqueous phases, upon which multiscale heterogeneous hydrogels can further be constructed and tailored for various applications.

### Enhanced interfacial mechanics in TAP gels

**Fig. 4:**
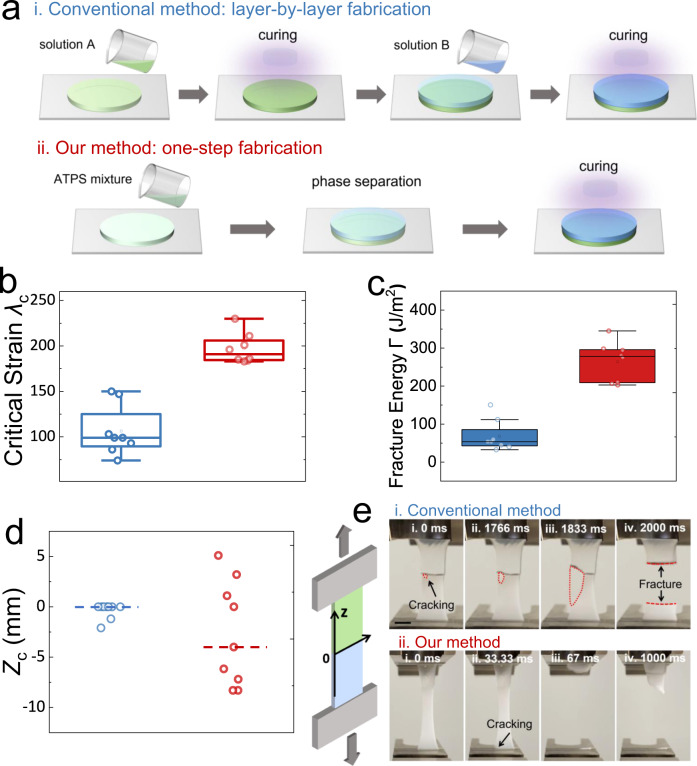
Enhanced interfacial mechanics of TAP gels. **a** Schematics of the conventional method and our method in constructing heterogeneous hydrogels with two layers. Comparison of critical strain ${\lambda }_{c}$ (**b**), fracture energy $\varGamma$ (**c**), and fraction position ${Z}_{c}$ (**d**) of TAP gel 1 constructed by the conventional method (colored in blue, ${\lambda }_{c}=106\pm 25.8\%$, $\varGamma=68.4\pm 38.3\,{{{\rm{J}}}}/{m}^{2}$, ${Z}_{c}=-0.37$) and our method (colored in red, ${\lambda }_{c}=197\pm 15.5\%$, $\varGamma=264.2\pm 48.5\,{{{\rm{J}}}}/{m}^{2}$, ${Z}_{c}=-2.7$). The box in **b** and **c** represents the middle 50% of the data, with the lower and upper bounds of the box being the 25th and 75th percentiles, respectively. Whiskers extend from the box to the minimum and maximum values that are not considered outliers. *n* = 8 independent samples in (**b**, **c**). The dashed lines in **d** represents mean values (*n* = 9 independent samples). **e** Distinct fracture dynamics observed in TAP gel 1 fabricated by the conventional method and our method. The scale bar is 1 cm. The hydrogel is comprised of 10 wt% PEG, 10 wt% DEX, and 10 wt% monomer-acrylamide. The weight of the covalent crosslinker, MBAA, is at 0.25 wt% of the monomer, and the photo-initiator is at 0.5 wt% of the monomer.

A conventional layer-by-layer approach has been widely used to fabricate heterogeneous hydrogels with multiple layers at macroscales (Fig. 4a.i). In the present study, we have compared the conventional approach with our ATPS-based one-step fabrication method. In general, the ATPS-based method is simpler and more efficient as it allows a one-step fabrication of multi-layer heterogeneous hydrogels compared to the conventional sequential approach. For example, a hydrogel with multilayers can be constructed in a single step of solution preparation and crosslinking, while the conventional method requires tediously repeating similar procedures many times in a layer-by-layer manner (Fig. 4a). Supplementary Table 2 is included to estimate the saved time by our method compared to the conventional method, which reveals that our method could be more efficient for fabricating heterogeneous gels with multiple layers (large k) or gels that take hours or days to crosslink (large *t*), such as PVA gels.

Moreover, we anticipate that the heterogeneous hydrogels fabricated from our approach may exhibit enhanced interfacial mechanics. As the crosslinking occurs uniformly at both phases when they are still in the liquid state, better connections of different gel phases at the interface may be achieved. To prove the hypothesis, we fabricate TAP gel 1 with the above two different methods and compare their mechanical properties. Several critical parameters, including critical strain ${\lambda }_{c}$ (the maximum stain before fracture) and fracture energy $\varGamma$ (the area under the stress–strain curve), are compared (Supplementary Fig. 15). Six hydrogel samples are tested for each method to minimize errors. Compared with the conventional method, heterogeneous hydrogels fabricated by our approach show enhanced mechanical properties, where the ${\lambda }_{C}$ and $\varGamma$ are increased two and four times, respectively (Fig. 4b, c). Interestingly, the heterogeneous hydrogels fabricated by these two methods show comparable mechanical moduli (slope of the stress–strain curve) (Supplementary Fig. 15), which suggests that different fabrication methods do not significantly affect the mechanical properties of hydrogels until they approach the point of fracture. In addition, it is worth noting that TAP gel 1 fabricated by the conventional layer-by-layer method always starts breaking at the interface, while the hydrogels fabricated by our one-step fabrication method seem to fracture at random positions (Fig. 4d). Such observation indicates that the interface of TAP gel 1 fabricated by the conventional approach is weaker than the rest parts of the hydrogel and thus start breaking earlier. The small cracks formed at the interface could nucleate stress and propagate, which eventually lead to a catastrophic network failure^68^.

This remarkable mechanical performance at the interface enabled by our method is a prominent point and may be qualitatively explained by the formation of a stronger network across the interface. In the conventional method, because a monomer solution is cured on the solid surface of a pre-formed hydrogel, the strength of the interfacial connection strongly depends on how much monomers can penetrate the pre-formed hydrogel. Therefore, due to an intrinsic concentration gradient of monomers at the interface, it is difficult to form a uniform hydrogel network across two gel phases. To further enhance the interfacial connection, it may be necessary to combine additional treatments, such as topological adhesion^69^ and ultrasound cavitation^25^, with the conventional method, which makes the manufacturing process more complicated and time-consuming. In contrast, our method allows different hydrogel layers to be “loaded” into ATPS, which means the hydrogel networks can be more evenly formed across the interface, thus enhancing the interfacial mechanics. Consequently, TAP gels fabricated by this approach could dissipate the stress more homogeneously across the polymer network, which may account for an increase in the critical strain and fracture energy observed in experiments.

Furthermore, the heterogeneous hydrogels fabricated by two methods exhibit distinct fracture dynamics. The fracture area grows slowly over time until a complete breakup (~2000 ms), as observed in the hydrogel fabricated by the conventional method (Fig. 4e). This slow fracture behavior is akin to the peeling adhesives from surfaces, a phenomenon commonly characterized by the inherent weakness of interfacial connections in the absence of further modifications^43,70^. In contrast, hydrogels fabricated by our method exhibit a rapid fracture process that only lasts tens of milliseconds (Fig. 4e). Such rapid fracture behavior was often observed for covalently crosslinked PAM hydrogels with a homogeneous polymer network^14^. Since the polymer network is homogeneous, meaning that it can dissipate the stress uniformly across the whole network, the tensile stress (proportional to the applied stain) could be increased to a much higher value until the fracture occurs. The distinct fracture dynamics between hydrogels fabricated by the two methods further indicate their different interfacial mechanics, from which our method shows better performance.

### Programmable cell patterns within TAP gels by 3D printing

Fabricating embedded heterogeneous hydrogels at mesoscale scales in a programmable manner remains challenging in the conventional layer-by-layer approach. To solve this problem, extrusion-based 3D printing technology has been widely used to construct complex hydrogel architectures owing to its compatibility with diverse ink materials and nozzle designs^71^. Notably, ATPS has recently been integrated with 3D bioprinting for constructing 3D architectures in all-aqueous environment^53,72^. Through interfacial complexation by electrostatic interactions or hydrogen bonding, 3D architectures can be directly printed into an aqueous matrix using two aqueous phases of the ATPS^52,53^. Printed structures show selective permeability to facilitate compartmentalized chemical reactions^52^ and can hold their shape for days before they begin to collapse^53^. However, these aqueous structures are not expected to survive after severe mechanical forces that may originate from continuous flows in real applications, nor can’t sustain their morphologies for longer periods, such as days or months. Therefore, a forward-looking step that transforms aqueous structures into hydrogels by introducing a gelation process will be necessary and offer greater mechanical stability for heterogeneous hydrogel structures.

**Fig. 5:**
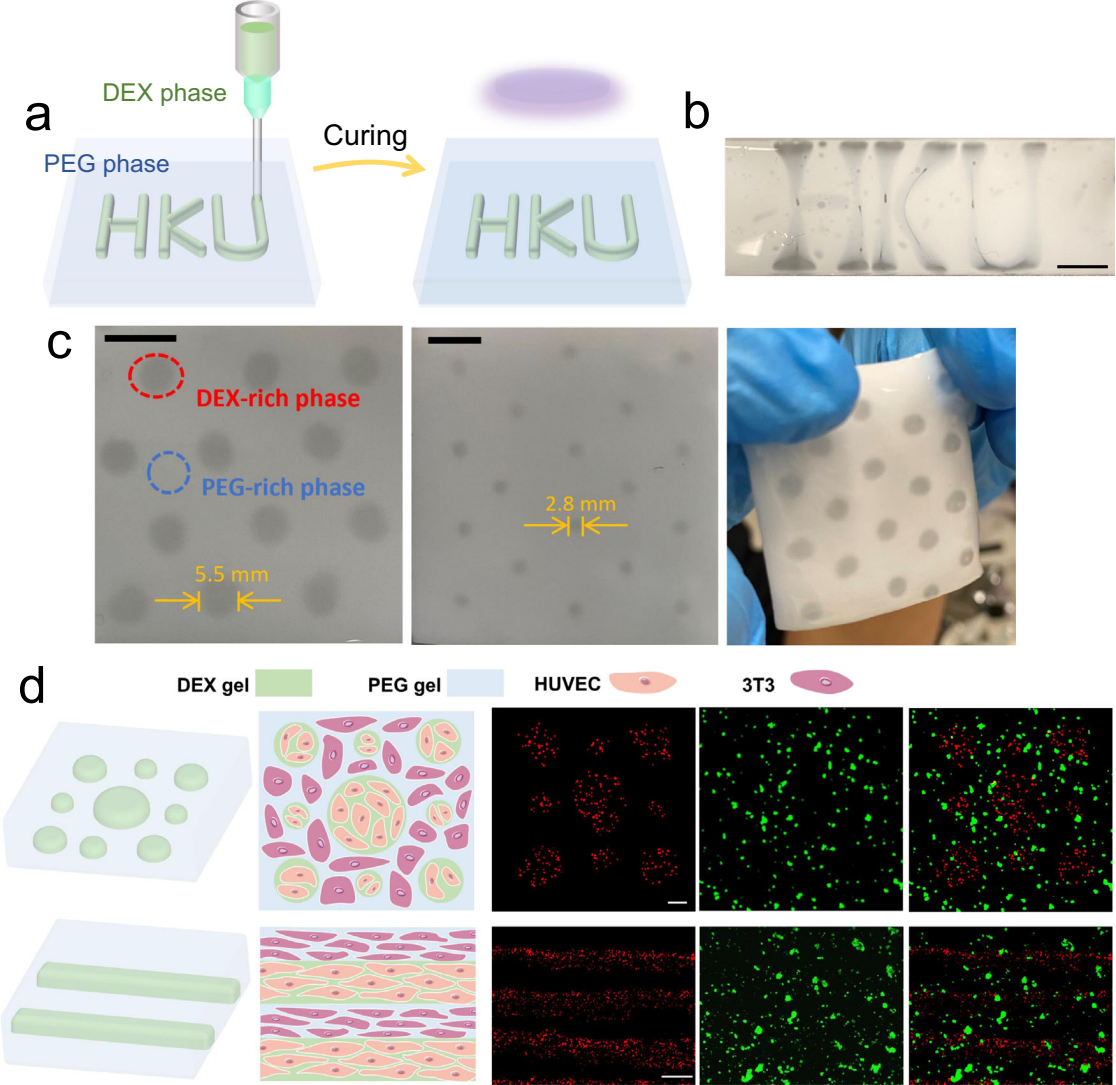
3D-printed cell patterns within TAP gels. **a** Schematic figures of constructing programable patterns within TAP gels by 3D printing. Photographs of printed heterogeneous TAP gels showing **b** “HKU” and **c** “dot” patterns with controllable sizes. Scale bars are 1 cm. **d** Cell patterns constructed within TAP gels. Spherical and linear DEX gel phases containing HUVEC (red fluorescence signal) are printed within the PEG gel phase containing 3T3 (green fluorescence signal). Scale bars are 500 µm.

To prove the concept, we propose that sophisticated architectures can be firstly built in an all-aqueous environment by 3D printing using DEX aqueous phase as the ink and PEG aqueous phase as the matrix (Fig. 5a). Upon the completion of the printing process, the heterogeneous architectures are stabilized by crosslinking the matrix and ink simultaneously into hydrogels (Fig. 5a). Two patterns, “HKU” and “dots” are constructed as examples where the embedded DEX gel shows higher transparency than surrounding PEG gel, which is consistent with our results in the previous section. Especially by controlling flow rates of the ink and printing speeds of the nozzle, the structure and size of the printed architecture can be further tuned in a tailor-designed manner (Fig. 5c). The formed heterogeneous hydrogel can sustain its morphologies for days and months if in a hydrated environment.

To further mimic cell collections and patterns in biological tissues, we anticipate directly printing cell patterns within TAP gels. To create a biocompatible environment, poly(ethylene glycol) diacrylate (PEGDA) and lithium phenyl-2,4,6 trimethylbenzoylphosphinate (LAP) is used as the monomer and photo-initiator due to their good biocompatibility. Two types of cells, human umbilical vein endothelial cells (HUVEC) and NIH 3T3 cells (3T3) are mixed with aqueous DEX phase and PEG phase as the ink and matrix, respectively. Cell patterns are printed within an aqueous environment in a programmable manner, followed by crosslinking under a blue light for seconds (Fig. 5a). Firstly, dome-like DEX gel phases containing HUVEC are spatially positioned within a bulk PEG gel phase that contains 3T3. Meanwhile, the size of the DEX gel phase can be tuned by controlling the printing speed of the nozzle. In addition, linear DEX gels with HUVEC are constructed parallel to each other, surrounded by a bulk PEG gel phase that contains 3T3. These embedded heterogeneous structures resemble cell patterning and organizations observed in many biological tissues, such as human skin.

Additional experiments are performed to prove the biocompatibility of TAP gel for the long-term survival of cultured cells. Results reveal that cells have more than 95% viability in the DEX gel phase after 7 days, whereas the PEG phase only has around 60% viability after the same period of time (Supplementary Fig. 16). Despite good viability, cells do not show good spreading morphologies in both gels and even tend to aggregate in the PEG phase (Supplementary Fig. 16). In future studies, the spreading of cells on polymer matrices could be enhanced by adjusting mechanical properties of hydrogels or functionalizing hydrogel polymer backbones with cell-adhesive motifs^73^. The above experiments demonstrate that TAP gels can be constructed in a programable manner by 3D printing, such as being useful in constructing sophisticated synthetic tissues by culturing cells in different domains.

### Selective partitioning and release of molecules using TAP gels

Another hallmark of biological systems is the ability to compartmentalize biomolecules for regulating biological functions^5,6^. The concept of compartmentalization has recently gained significant attention in different scientific communities due to the discovery of membraneless organelles, also known as biomolecular condensates, in cells^46^. For example, cells can take advantage of phase separation to form biomolecular condensates for regulating several biological functions, such as cell signaling, gene regulation, and transcription^5,6,74^. Therefore, we have also explored the compartmentalization ability of TAP gels, which may facilitate a heterogeneous distribution of molecules at the microscale.

**Fig. 6:**
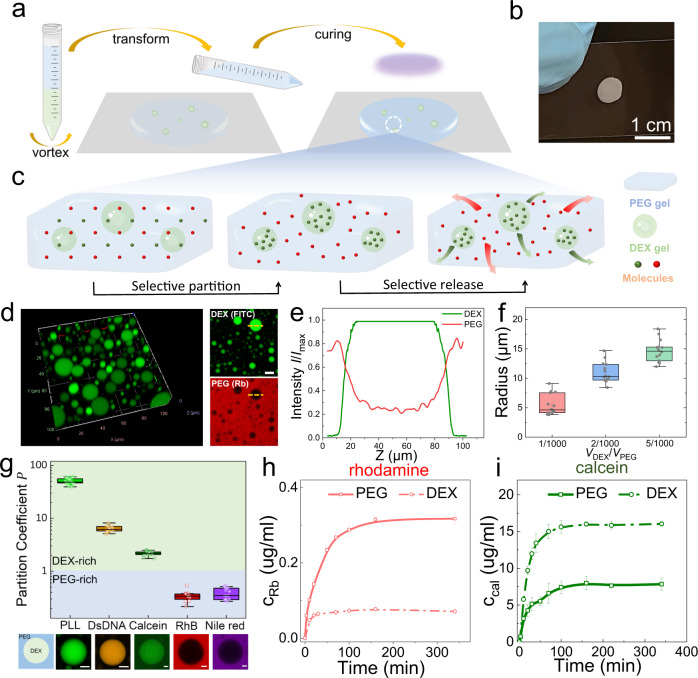
Selective partitioning and release of molecules using TAP gels. **a** Schematic illustration of fabrication procedures of the TAP gel with DEX microgels embedded within a PEG gel. Due to their different affinities with aqueous phases, molecules preferentially partition into different phases. **b** Macroscopic picture of a thin film made of the heterogeneous TAP gel with microgels embedded. **c** Schematic diagram of selective partitioning and release of molecules within the TAP gel. **d** Confocal microscopic images of spherical DEX microgels distributed in the PEG gel. The scale bar is 20 µm. **e** Relative fluorescence profile *I*/*I*_max_ indicating the distribution of DEX (FITC-labeled) and PEG (Rh-labeled) within and out of the droplets. *I*_max_ represents the maximum value of fluorescence intensity in the absorption unit (a.u.). **f** Controllable size distribution of DEX microdroplets within the PEG phase measured by DLS. *n* = 3 independent samples. **g** Partition coefficients of different molecules in ATPS indicated by the ratio of fluorescence intensity in and out of droplets. Scale bars are 20 µm. Results are collected from more than *n* > 20 different droplets to calculate the partition coefficient $P.$ The box in **f** and **g** represents the middle 50% of the data, with the lower and upper bounds of the box being the 25th and 75th percentiles, respectively. Whiskers extend from the box to the minimum and maximum values that are not considered outliers. Release profiles of Rhodamine **h** and calcein **i** from the PEG and DEX gel, respectively. Error bars indicate mean ± SD (*n* = 3 independent samples).

Firstly, we create microgels embedded in another bulk hydrogel using a vortex-assisted emulsification method (Fig. 6a). A turbid mixture comprised of aqueous PEG and DEX phases at different ratios is poured onto a substrate and further crosslinked into a hydrogel thin film (Fig. 6b). Under this construction, spherical DEX microgels are randomly embedded in the three-dimensional space of the bulk PEG hydrogel, as characterized by CLSM (Fig. 6d). A phase transition from DEX (FITC labeled) to PEG (Rh labeled) is also revealed by the distribution of fluorescence signals in and out of droplets (Fig. 6e). In addition, the size distribution of spherical DEX gels dispersed in PEG bulk phase can be controlled by readily adjusting the volume ratio (${V}_{{{\rm{DEX}}}}/{V}_{{{\rm{PEG}}}}$) between the DEX and PEG phases as revealed by Dynamic light scattering (DLS) (Fig. 6f). We further confirm it by fluorescence microscope imaging to obtain the droplet size distribution of spherical DEX gels. Results show that with the increase of ${V}_{{{\rm{DEX}}}}/{V}_{{{\rm{PEG}}}}$, the peak of size distribution histograms shift to large values (Supplementary Fig. 17), which is in good agreement with the DLS results.

Due to intrinsic affinities and the lack of a physical membrane between two aqueous phases, molecules can preferentially partition and locally concentrate in one of the two phases (Fig. 6c). The partition affinity depends on their physicochemical properties, such as molecular weight, hydrophobicity, and interaction motifs^75^. To prove the selective partitioning, we test the partition coefficient of several molecules, as quantified by the ratio of fluorescence intensity between two phases ($P={I}_{{{\rm{DEX}}}}/{I}_{{{\rm{PEG}}}}$). It is shown that biomolecules and synthetic polymers, such as Poly-l-lysine (PLL), double-strand DNA (DsDNA), and calcein prefer to partition into the DEX phase (Fig. 6g). Especially, PLL shows the highest selective partitioning ability to the DEX phase with a partition coefficient $P$ = 50.1 ± 5.8. However, some hydrophobic molecules, such as Rhodamine 6 G and Nile red, prefer to stay in the PEG phase. Thus, their partition coefficients are smaller than 1 (Fig. 6g). Relevant information about used molecules, including molecular weight (Mw), charge at neutral PH, and partition coefficient ($P$), are summarized in Supplementary Table 3. In addition, it is expected that monomers used in this work to form hydrogel networks could show selective partitioning between two phases. For example, it is revealed that both storage and loss moduli of the PEG gel phase are one order magnitude larger than those of the DEX gel phase in TAP gel 3, as characterized by a rheometer (Supplementary Fig. 18). This concurs with a preferred partitioning of PEGDA monomers into the PEG-rich phase due to their similar chemical structures before the crosslinking, thereby forming a denser polymer network in the PGE-rich phase after the crosslinking. However, it is technically challenging to quantify the accurate partition coefficient of monomers due to difficulties in the selective labeling of monomers. Therefore, the uneven distribution of monomers into two phases could enable the construction of heterogeneous hydrogels with two gel phases showing different physical and chemical properties.

Moreover, TAP gels could allow the simultaneous release of different molecules with a spatiotemporally controlled release profile (Fig. 6c). As a demonstration, we characterize the release of Rhodamine and calcein from different gel phases of TAP gels after their selective partitioning. By immersing PEG and DEX gels in water separately, encapsulated molecules gradually diffuse to the surrounding water, and their real-time concentration is monitored by UV–vis microscopy. The PEG gel is shown to release Rhodamine at a faster speed and a higher saturation concentration compared to the DEX gel (Fig. 6h). In contrast, the DEX gel can release calcein faster and more than the PEG gel. In addition to small molecules, large polymers can also selectively release from TAP gels, such as FITC-DEX and Rh-PEG (Supplementary Fig. 19). The difference is that small molecules release faster than large polymers, which seems reasonable because large polymers have smaller diffusion coefficients compared to small molecules. The different release profile of molecules from different gel phases is an inherited nature of their selective partitioning, and their preferential release from specific gels is reconciled with their measured partition coefficients (Fig. 6g). Therefore, it is possible that multiple molecules can be released within TAP gels in a spatiotemporally controlled manner when further integrated with programable techniques, such as 3D printing. The selective partitioning and release of molecules in TAP gels provide a versatile platform for various applications, such as wound dressing and tissue regeneration^8^.

## Discussion

In this work, we have demonstrated a nature-inspired approach based on phase separation to construct heterogeneous TAP gels at different length scales, mimicking biological systems in key features, such as muscle-tendon connections, cell patterning, and molecular compartmentalization. Beyond its simplicity and efficiency, our one-step fabrication method enables the construction of sophisticated embedded structures that are nevertheless difficult to be constructed by conventional methods. We have demonstrated that the physiochemical properties of each phase in TAP gels, such as stiffness, stretchability, and affinity to certain molecules, can be readily controlled by changing polymer compositions and gelation conditions. Moreover, TAP gels fabricated by our one-step approach exhibit enhanced interfacial mechanics compared with their counterparts obtained from conventional layer-by-layer methods. We attribute this enhancement to the increased penetration and connection of the polymer network across the interface. This approach represents a facile strategy to enhance the interfacial connection between different gel phases, in addition to some previously reported methods, including surface modification^24,70^, ultrasound cavitation^25^, and topological adhesion^69^. Notably, the last two methods enhance the interfacial connection by facilitating the diffusion of monomers into the preformed hydrogels before crosslinking the second phase, which could be similarly achieved using our approach.

Our methodology of fabricating TAP gels should be applicable to a wide range of materials, including biopolymers, synthetic polymers, multivalent ions, and surfactants^49,50^. In this work, we have selected different materials sources, including biopolymers (DEX and sodium alginate) and synthetic polymers (PEG, PEGDA, and PVA), to demonstrate the universality of our fabrication method. It is worth noting that the majority of materials demonstrated here have been approved by regulatory agencies, including the Food and Drug Administration (FDA) in the United States, so they could be regarded as candidate materials for food and biomedical applications.

Heterogeneous TAP gels may benefit a plethora of applications requiring tissue-like properties. For instance, distinct physicochemical properties of extracellular matrices can significantly affect cells migration and proliferation^7,76–79^. TAP gels that provide heterogeneous structures and physicochemical properties could serve as an enabling platform mimicking extracellular matrices for investigating physiological and pathological cell dynamics in vitro, including embryogenesis, wound healing, and cancer metastasis^80–82^. In addition, in the case of mimicking biological muscle-tendon connections, the combination of soft and stiff hydrogels could be deployed to design bridging materials connecting soft biological tissues and rigid electronic devices with desirable affinity for health monitoring applications^9^. Lastly, the property of selective partition and release of molecules within TAP gels may be beneficial for designing drug carriers with controlled encapsulation and release properties. Such properties are desirable for applications in wound dressing, healing, and tissue engineering applications^4,8,83^.

## Methods

### Materials

Poly(ethylene glycol) (PEG, Mw ~8000), Poly(vinyl alcohol) (PVA, Mw: 89,000–98,000, 99% hydrolyzed), Poly(2-ethyl-2-oxazoline) (PEtOx, Mw~50, 000), sodium citrate, Poly(ethylene glycol) diacrylate (PEGDA, Mn~575), sodium alginate, 2-Hydroxy-4′-(2-hydroxyethoxy)−2-methylpropiophenone (Irgacure 2959), N,N′-methylenebisacrylamide (MBAA), were purchased from Sigma-Aldrich. Dextran (DEX, Mw ~10,000, 40,000, and 500,000) and Nile red were purchased from Macklin. Acrylamide and lithium phenyl-2,4,6 trimethylbenzoylphosphinate (LAP) were purchased from TCI. Rhodamine 6 G, Fluorescein isothiocyanate (FITC) labeled Poly-l-lysine (FITC-PLL, Mw ~30,000–70,000), and Fluorescein isothiocyanate–dextran (FITC-dextran, Mw ~40, 000) were purchased from Sigma-Aldrich. Rhodamine-labeled PEG (Rh-PEG, Mw ~40,000) was purchased from Aladdin. Nucleic acid sequences of Cy5-DNA were synthesized by Integrated DNA Technology (IDT). Calcein was purchased from J&K scientific. Millipore Milli-Q water (18.2 MΩ, pH = 7) was used in all experiments. All materials were used as received without further purification.

### Multilayer gel fabrication

Polymers were first dissolved into Milli-Q water (18.2 MΩ, pH = 7) to induce phase separation at certain molecular weights (Supplementary Table 1). The turbid solution was then centrifuged at 4000*g* for 5–30 min to reach an equilibrium. Immiscible phases were extracted and collected into separated tubes in sequence. Then, the crosslinker and photo-initiator were dissolved into collected solutions at a specific weight ratio of monomer (Supplementary Table 1). For TAP gels, two prepared aqueous phases were simultaneously added into sealed fabrication molds and a liquid–liquid interface due to the immiscibility of two phases. Then, the mold was exposed to UV light for 15 min. After the curing step, TAP gels were taken out for further characterizations. As a comparison to TAP gels, a hydrogel with similar two layers were fabricated following a layer-by-layer method. Firstly, one aqueous solution with dissolved crosslinkers and photo-initiator was added into sealed fabrication molds and completely crosslinked under UV light for 15 min. Then, the second solution was added on the top of crosslinked first hydrogel and wait for 15 minutes to allow monomers in the second solution to diffuse as much as possible into the pre-formed first hydrogel. We then expose the mold under UV for another 15 min to form the second hydrogel layer with a connection to the first one.

### TAP gel with embedded micro-gels

The ATPS was firstly prepared by dissolving 10 wt% PEG 8000, 10 wt% Dextran T10, and 10 wt% PEGDA (Mn~575) into Milli-Q water (18.2 MΩ, pH = 7). The solution was then centrifuged at 8000 rpm for 5–30 min to reach an equilibrium. Two immiscible phases, PEG and Dextran phases, were collected into separated tubes in sequence. Then, we added LAP, at 0.5 wt% of the weight of PEGDA, as the photo-initiator. Two prepared PEG and DEX phases were added into a microcentrifuge tube at varying volume ratios and then vortexed for seconds. The turbid solution was transferred into a fabrication mold and the mold was exposed to a blue light for seconds. After the curing step, hydrogels were taken out for further characterizations.

### Characterization

Optical, fluorescence and 3D images were captured by a confocal laser scanning microscope (CLSM, Carl Zesis LSM 700) and analyzed using the ZEN software. All plots and charts were drawn using Origin 2021. Microscale morphologies of hydrogels were captured by Scanning Electron Microscope (SEM, Hitachi S4800 FEG). All SEM specimens were firstly prepared through a freeze-drying method. Hydrogel samples were completely frozen at −80 °C freezer for one day. After the pre-freezing process, hydrogel samples were transferred into a freeze dryer (FD-1D-50, BIOCOOL) and kept there more than 2 days. The dried hydrogels were cut to expose cross-sections after plunge-freezing into liquid nitrogen. Samples were then sputtered with gold nanoparticles (thickness <20 nm, Quorum Q150T Plus ES) for around 30 s in vacuum ( ~ 5 × 10^−5^ mbar). Energy dispersive X-ray (EDX, Hitachi S4800 FEG) was used to obtain the elemental mapping on cross-sections. In addition, critical point drying (CPD, Tousimis Autosamdri 931) method was also tried for hydrogel samples but proved not applicable in this work because hydrogels significantly shrunk, became less transparent, and consequently behaved no clear porous structures after CPD (Supplementary Fig. 20). Before the CPD, hydrogel samples were immersed in the graded ethanol solution (50%, 75%, 88%, 100% v/v, respectively) in a stepwise manner by gradually adding absolute ethanol into the solvent every 12 h. Hydrogel samples were completely dehydrated after 2 days and were immediately taken out for performing CPD. The dried samples were stored in a desiccator to prevent rehydration.

### Mechanical testing

A mechanical testing machine (ZwickRoell) was used for the tensile testament of TAP gels. The width and thickness of dumbbell-shaped hydrogels were measured using a caliper and typed into the ZwickRoell software. Specimens were tested at a 50 %/min strain rate while stress-strain curves were automatically obtained by the software. All data were further analyzed to calculate the modulus, critical strain, and fracture energy of hydrogels. For each TAP gel, at least three independent samples have been fabricated and characterized for getting an average value as mean ± standard deviation (SD).

### Cell culture in TAP gels

ATPS were firstly prepared by dissolving 10 wt% PEG, 10 wt% Dextran, 10 wt% PEGDA and 0.5 wt% LAP in Hank’s balanced salt solution (HBSS, no calcium, no magnesium, no phenol red, ThermoFisher). The upper PEG phase and lower DEX phase were then obtained after a complete phase separation. For cell patterning experiments, HUVEC cells and 3TE cells were stained using different trackers (DiD Perchlorate, excitation/emission λ 644/665 nm; DiO Perchlorate, excitation/emission λ 483/501 nm, Yeasen, China). The stained cells were then mixed with DEX and PEG phases, respectively. Using a 3D printer (Ultimaker 2+, Netherlands), the DEX ink phase, was directly printed within the PEG bulk phase. A low ink flow rate (0.2–1 mL/h) was used to ensure good cell viability. The printed patterns were then crosslinked under blue light (405 nm) for 10 s.

For cell viability experiments, HUVEC cells were mixed with PEG and DEX phases and transferred to a 48 well plates at a volume of 80 μL per well. Solutions were crosslinked under blue light for around 10 s to make sure gels in all wells were formed. Gels with cells were cultured in a medium prepared with 90 % v/v Dulbecco’s Modified Eagle Medium (DMEM, ThermoFisher) and 10% v/v Fetal Bovine Serum (FBS, ThermoFisher) and transferred to CO_2_ incubator at 37 °C. Culture medium were refreshed every 2 days. Cell viability was measured by staining cell using a LIVE/DEAD Viability/Cytotoxicity kit (ThermoFisher). The number of cells were counted using the ImageJ software (National Institutes of Health, USA). Results were collected on day 1, day 5, and day 7.

### Size distribution measurement

The size distribution of DEX microdroplets was measured through dynamic light scattering (DLS) using Zetasizer Pro (Malvern Instruments, UK). ~1 mL sample was pipetted into a transparent cuvette (DTS0012) and stored into the machine. The viscosity of water (1.0 mPa) and the refractive index of DEX (1.38) was used in the program. The measurement was conducted at room temperature with a 120 s equilibration time. Five times measurements were carried out for each sample and at least three independent samples were measured to get a mean ± SD.

### Releasing profile

ATPS, consist of PEG, DEX, PEGDA (monomer) and LAP (photo-initiator), containing Rhodamine (100 ug/ml), calcein (800 ug/ml), FITC-DEX (6 mg/ml), and Rh-PEG (8 mg/ml) were firstly prepared. Aqueous PEG and DEX phases were then separately extracted from equilibrated ATPS and crosslinked into hydrogel at a volume of 50 μL. The formed hydrogels were placed in a 2000 μL Milli-Q water solution. Encapsulated fluorescent molecules released from hydrogels into the bulk water driven by concentration gradients. The absorption intensity of the bulk water solution was measured over time by a microplate reader (Molecular Devices) and further calculated into relative concentrations of released molecule based on standard calibration curves (Supplementary Fig. 21).

### Porosity of hydrogel

To calculate the porosity of TAP gels, SEM images were processed with the image binarization by the ImageJ software (National Institutes of Health, USA) with certain thresholds (Supplementary Fig. 22). In binary images, the white area represents pores, and the black area represents polymer networks. Therefore, the porosity was calculated as the ratio of white area to the whole areas. At least three independent samples were processed and calculated to get a mean ± SD.

### Statistics and reproducibility

No statistical method was used to predetermine sample size. No data were excluded from the analyses. Each result presented in work was obtained after at least three independent experiments with similar results.

### Reporting summary

Further information on research design is available in the Nature Portfolio Reporting Summary linked to this article.

## Supplementary information

Supplementary Information

Reporting Summary

## Data Availability

All data needed to evaluate the conclusions in the paper are present in the paper and/or the Supplementary Information, and also from the corresponding author upon request. Source data are available in figshare database (10.6084/m9.figshare.22613260). Source data are provided with this paper.

## Source data

Source Data
