# Supplementary material for: Phase-separation facilitated one-step fabrication of multiscale heterogeneous two-aqueous-phase gel
Source: Nat Commun. 2023 May 16;14:2793. doi: 10.1038/s41467-023-38394-9 (PMC10188440; doi:10.1038/s41467-023-38394-9)
Supplement: Supplementary file 1 — Supplementary Information [file 41467_2023_38394_MOESM1_ESM.pdf]

Supplementary information for

Phase-separation facilitated one-step fabrication of multiscale heterogeneous two-aqueous-phase (TAP) gel

Feipeng Chen, Xiufeng Li, Yafeng Yu, Qingchuan Li, Haisong Lin, Lizhi Xu, Ho Cheung Shum*

*Corresponding author. Email: ashum@hku.hk

Supplementary Note 1: Flory-Huggins theory

The thermodynamic principles of phase separation can be understood by considering the free energy of the system: $\Delta f = \Delta \mathrm{H} - \mathrm{T}\,\Delta \mathrm{S}$, where $\Delta \mathrm{H}$ is the enthalpy change and $\Delta \mathrm{S}$ is the entropy change. Under the classic Flory-Huggins (FH) framework, Δf are typically expressed as functions of volume fractions of components ϕ_i and interaction parameters χ_{ij}. Phase separation occurs only when $\Delta f < 0$, which requires entropic contribution favoring mixing to be smaller than enthalpic contribution caused by incompatible interactions amongst polymers. The free energy Δf are contributed by entropy increment $\Delta \mathrm{S}$ favoring mixing and enthalpy penalty $\Delta \mathrm{H}$ attributed by repulsive molecular interactions, which are described as follows:

$$\Delta f = \Delta \mathrm{H} - \mathrm{T}\,\Delta S \qquad \text{(s1)}$$

$$\Delta S = \sum_i \frac{\phi_i}{N_i} \ln{(\phi_i)} \qquad \text{(s2)}$$

$$\Delta H = \sum_i \sum_{j=i+1} \chi_{ij}\phi_i\phi_j \qquad \text{(s3)}$$

where ϕ_i and N_i are volume fractions and polymer length (or degree of polymerization) of constituents. χ_{ij} is the FH interaction parameters which represents the strength of repulsive interactions between polymers. For uncharged species, equilibrium phases have same chemical potential $\mu_i^c = \partial f_i / \partial \Phi_i$. For more complicate systems, where charged polymers or salts are used as one of phases, there is extra electrochemical potentials need to be considered as $\Delta\mu_i^{el} = lnK_i$, where K_i is the partitioning coefficient and is Φ_i^t/Φ_i^t in ATPSs. Therefore, in a ATPSs system, the boundary conditions are as

$$\mu_{1,top}^c = \mu_{1,bottom}^c \qquad \text{(s4)}$$

$$\mu_{2,top}^c = \mu_{2,bottom}^c$$

Also, an incompressible condition is satisfied as,

$$\sum_i \phi_i = 1 \qquad \text{(s5)}$$

FH parameters χ_{ij} are usually given based on experiments. Then, equilibrium states can be numerically solved by minimizing the global free energy(*1*, *2*). Binodal curves are constructed by connecting all equilibrium points for distinguishing single-phase and two-phase region. The

binodal curves can be affected by many parameters, such as polymer length, PH, and temperature(*3*). These changes all lead to a varied FH parameter χ_{ij}. The polymer length is one of the mostly used parameters to control the position of binodal curves in the phase diagram. Typically, the phase separation will appear at lower concentrations when using polymers with higher polymer length. This can be perceived in Eq. (s2) where a higher polymer length gives a lower entropy contribution compared with the enthalpic contribution to the free energy and thus promote the phase separation. This theoretical analysis is consistent with our experimental observations in Supplementary Fig. 4 and Supplementary Fig. 5.

Supplementary Note 2: Determination of polymer compositions in PEG-rich and DEX-rich phases

The polymer ratios in each phase can be determined quantitatively through the phase diagram according to a lever rule[12]. For example, in Fig. R5, the yellow solid circle represents the initial polymer composition with x_0 DEX and y_0 PEG. Through the phase separation, the initial solution will demix along the tie line (black dash line) and reach two equilibrium states, the PEG-rich phase and DEX-rich phase, locating on the binodal curve. The polymer composition of PEG-rich and DEX-rich phase can be determined directly from the phase diagram according to respective values of x and y axis.

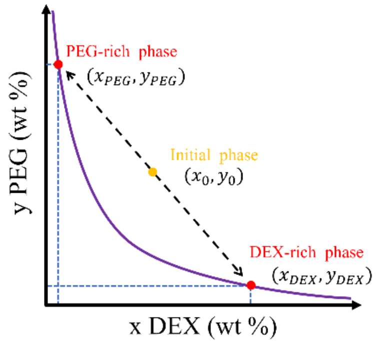

Supplementary Fig. 1. Schematic phase diagram showing polymer ratios of PEG and DEX in the initial phase, PEG-rich phase, and DEX-rich phase.

To exactly determine polymer compositions of two equilibrium states in our experiments, we assume polymer composition of PEG-rich phase, DEX-rich phase, and initial phase as (x_{PEG}, y_{PEG}) and (x_{DEX}, y_{DEX}), (x_0, y_0), respectively. According to mass and volume conservation laws, we have

$$x_{PEG}V_{PEG}\rho_{PEG} + x_{DEX}V_{DEX}\rho_{DEX} = x_0 V_0 \rho_0, \quad (1)$$

$$y_{PEG}V_{PEG}\rho_{PEG} + y_{DEX}V_{DEX}\rho_{DEX} = y_0 V_0 \rho_0, \quad (2)$$

$$V_0 = V_{PEG} + V_{DEX}. \quad (3)$$

Here, V and ρ are the volume and polymer density of different phases (PEG-rich, DEX-rich, and the initial solution). With the note widely confirmed by experiments that $\rho_{PEG} \approx \rho_{DEX} \approx \rho_0$,[13-15], above equations give to,

$$\frac{V_{PEG}}{V_{DEX}} = \frac{x_{DEX} - x_0}{x_0 - x_{PEG}} = \frac{y_0 - y_{DEX}}{y_{PEG} - y_0}. \quad (4)$$

Eq. (R4) indicates a lever rule that the volume ratio between the PEG-rich and DEX-rich equals the reverse ratio between distances of PEG-rich and DEX-rich to the initial phase in the phase diagram. Next, we experimentally calculate volumes of different phases and thus their ratio. We start with an initial volume $V_0 \approx 9.65 \pm 0.03$ ml with 10 wt% PEG and 10 wt% DEX (Fig. R6.a). After the phase separation, it is determined that $V_{DEX} \approx 2.16 \pm 0.04$ ml (Fig. R6.b), and thus $\frac{V_{PEG}}{V_{DEX}}$ is determined as ~3.47.

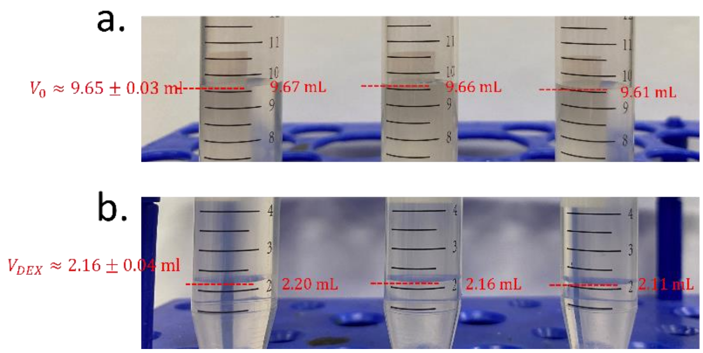

Supplementary Fig. 2. Photographs showing (a) total volumes as $V_0 \approx 9.65 \pm 0.03$ ml and (b) the volume of DEX-rich phase $V_{DEX} \approx 2.16 \pm 0.04$ ml for calculating the respective volume raito, $\frac{V_{PEG}}{V_{DEX}}$.

With the obtained volume ratio, we can determine polymer positions of PEG-rich and DEX-rich phases by drawing a tie line crossing the initial phase and intersecting the binodal curve with two points according to Eq. (R4) (Fig. R7). It shows that the PEG-rich phase contains 2.6 wt% DEX and 14 wt% PEG. In contrast, DEX-rich phase comprises 24.8 wt% DEX and 2.2 wt% PEG.

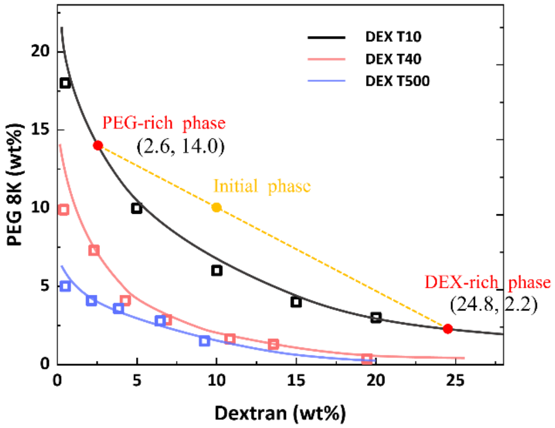

Supplementary Fig. 3. Experimentally obtained phase diagram for determining the polymer ratios in PEG-rich phase and DEX-rich phase in our experiments.

Supplementary Table 1: Polymer compositions and crosslinking conditions of TAP gels.

	ATPS composition	monomer	crosslinker	Photo-initiator
TAP gel 1	PEG 8000 (10 wt%), Dextran T10 (10 wt%)	Acrylamide (10 wt%)	MBAA (0.06-1 wt% of monomer)	Irgacure 2959 (0.5 wt% of monomer)
TAP gel 2	PEG 8000 (10 wt%), Dextran T10 (10 wt%), sodium alginate (1.25 wt%)	Acrylamide (10 wt%)	MBAA (0.25 wt% of monomer)	Irgacure 2959 (0.5 wt% of monomer)
TAP gel 3	PVA 8000 (10 wt%), Dextran T10 (10 wt%)	Acrylamide (10 wt%)	MBAA (0.25 wt% of monomer)	Irgacure 2959 (0.5 wt% of monomer)
MAP gel	PEG 8000 (10 wt%), Dextran T10 (10 wt%), PEtOx (10 wt%)	Acrylamide (10 wt%)	MBAA (1 wt% of monomer)	Irgacure 2959 (0.5 wt% of monomer)
TAP gel with embedded microgel	PEG 8000 (10 wt%), Dextran T10 (10 wt%)	PEGDA (Mn~575, 10 wt%)	—	LAP (0.5 wt% of monomer

Supplementary Table 2: Estimated time for fabricating heterogeneous hydrogels by the conventional method and our method.

Steps	Time (min, conventional method)	Time (min, our method)
Phase separation	——	5 min at 3000g centrifugation speed
Crosslinking	k·t	t
Total	k·t	t+5

*k: the number of layers
*t: crosslinking time per time

Supplementary Table 3: Relevant information about partitioned molecules, including molecular weight (Mw), charge at neutral pH, and partition coefficient.

Molecule	Mw (Da)	Charge (pH=7)	$P = \frac{I_{DEX}}{I_{PEG}}$*
FITC-PLL	30,000-70,000	Positive	50.1±5.8
Cy5-DNA	~13000	Negative	6.4±0.8
Calcein	622.6	Negative	2.2±0.2
Rhodamine 6G	479.0	Positive	0.34±0.07
Nile red	318.4	Neutral	0.38±0.09

*P: partition coefficient

I: fluorescence intensity of targe molecules

Supplementary Figures

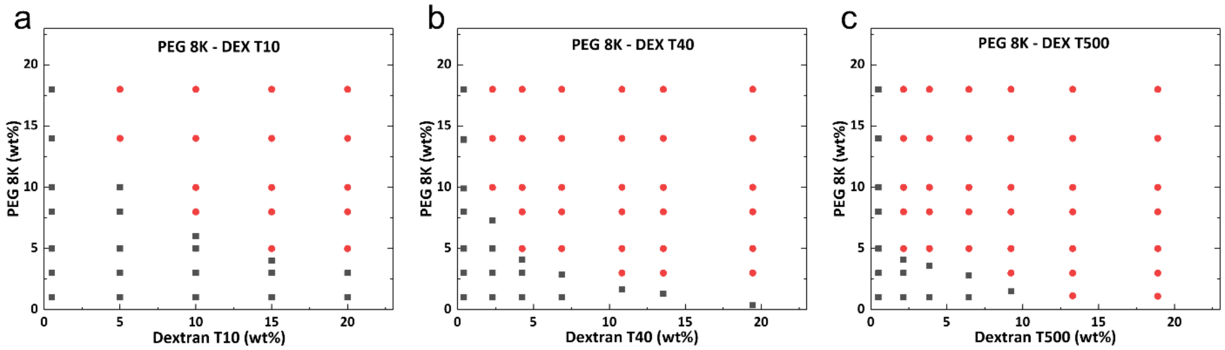

Supplementary Fig. 4: Phase diagram of ATPS consist of PEG 8K and DEX with different molecular weight: (a) DEX T10; (b) DEX T40; (c) DEX T500. The dark square represents single-phase regime while red circle represents the two-phase regime.

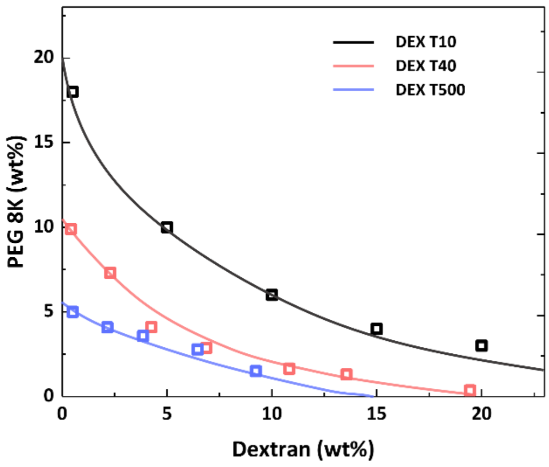

Supplementary Fig. 5: Binodal curves of ATPS consist of PEG 8K and DEX with different molecular weight: (a) DEX T10; (b) DEX T40; (c) DEX T500. The binodal curve shifts to bottom left corner as the molecular weight of DEX increase.

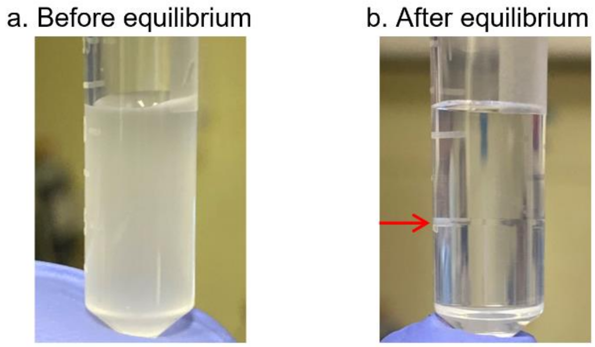

Supplementary Fig. 6: Macroscopic picture of ATPS before phase separation (a) and after phase separation (b). The mixture is turbid before the phase separation. After the phase separation, the mixture separates into two transparent and immiscible phases with an interface at the middle marked by the red arrow.

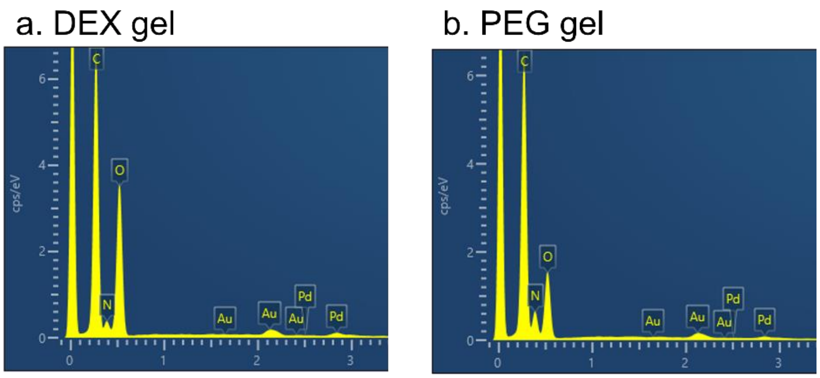

Supplementary Fig. 7: EDX elemental mapping analysis for the (a) DEX gel and (b) PEG gel in Fig. 2j, which shows the intensity of N element is very low, when compared to C and O elements.

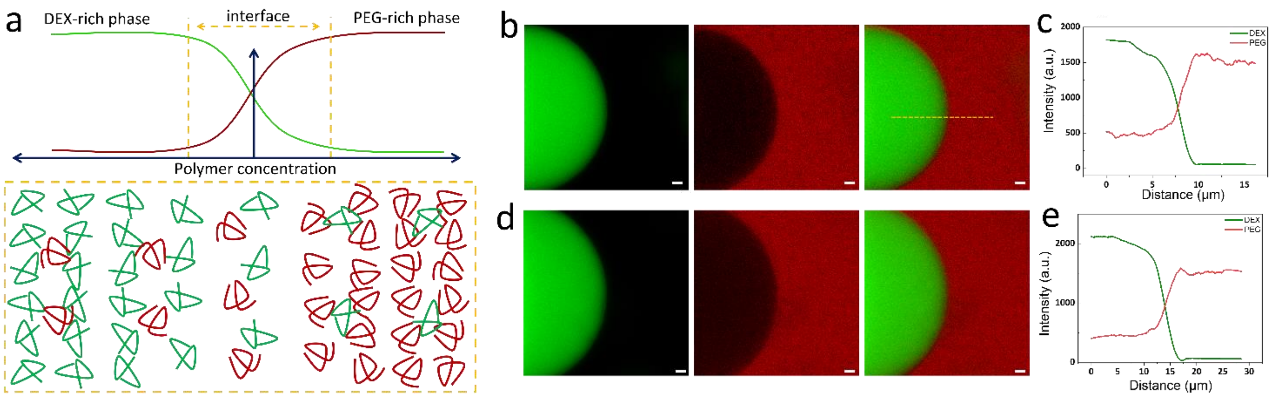

Supplementary Fig. 8: a) Schematic illustration of polymer distributions across the interface. (b) (d) Fluorescence images and (c) (e) intensity profiles of PEG and DEX which reveal polymer distributions around the interface. The unit of a.u. in (c) and (e) represents absorption unit. Scale bars in (b) and (d) are 2 μm.

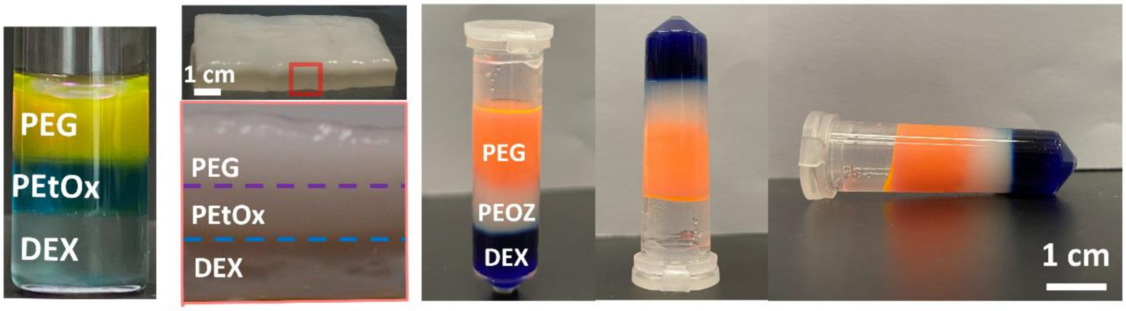

Supplementary Fig. 9: A3PSs-hydrogel consist of PEG, DEX, and PEtOx crosslinked by a polyacrylamide network. Three layers are colored with different dyes to show manifest different phases and interfaces in between.

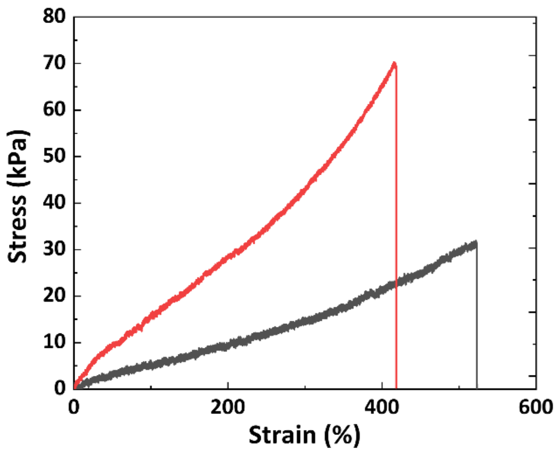

Supplementary Fig. 10: A representative stress-strain curve of TAP gel 1 hydrogel. The load is applied at a constant 50%/min strain rate while the curve is measured automatically by the software. The red curve is DEX gel and the black curve is PEG gel.

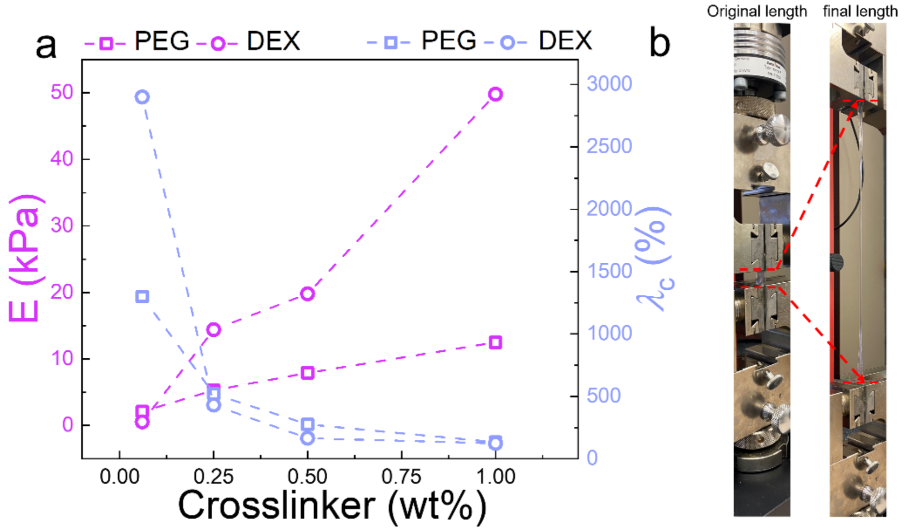

Supplementary Fig. 11: (a) Modulation of Young Modulus E and critical strain λ_c of PEG and DEX gels at different concentrations of crosslinkers. The Young Modulus E of both gel phases increases with an increasing crosslinker concentration. The stretchability of both gels decreases as the crosslinker concentration increases. (b) Highly stretchable DEX hydrogel that can be stretched to ~30 times longer than its original length at a very low concentration of crosslinker (0.06 wt%) of acrylamide.

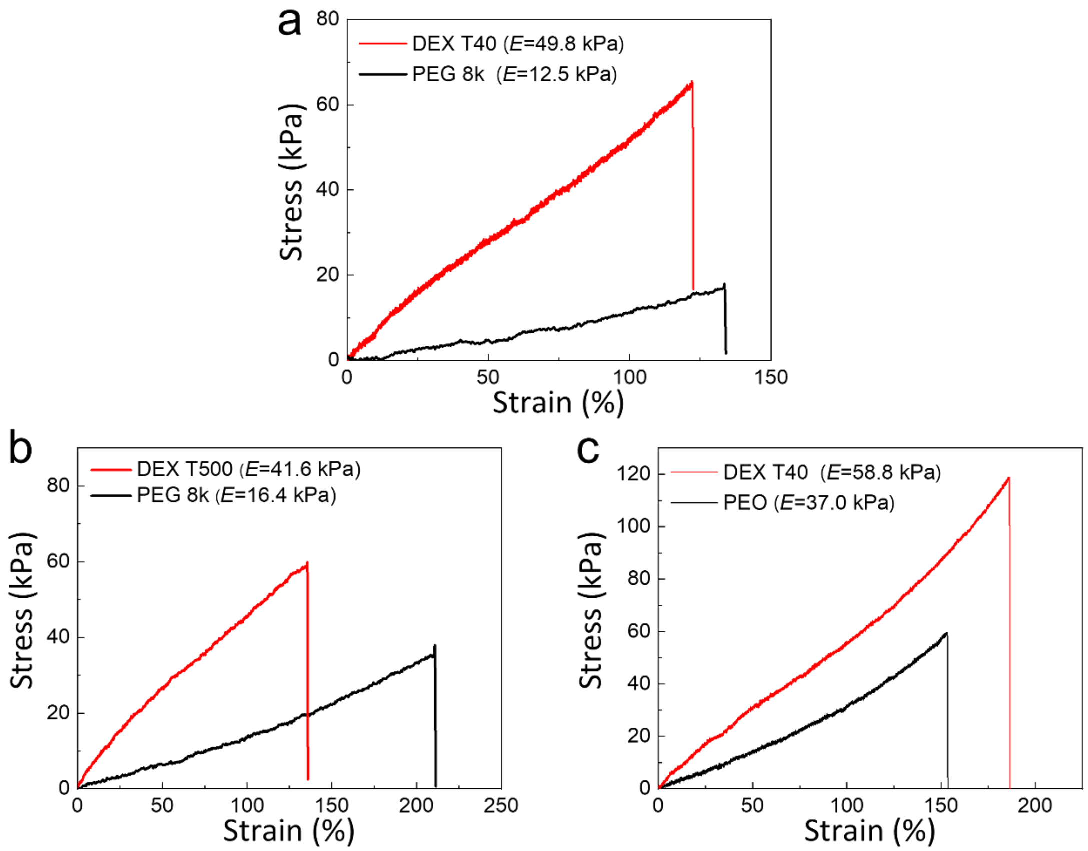

Supplementary Fig. 12: Comparison of mechanical properties of TAP gel with various polymer compositions. All TAP gel are comprised 10 wt% polymers for forming ATPS and 10wt% acrylamide as monomers, which are crosslinked with bis-acrylamide, at 1 wt% of the weight of acrylamide and Irgacure 2959, at 0.5 wt% of the weight of acrylamide, under UV light. Mechanical properties of TAP gel have not been clearly enhanced by using higher molecular weight of polymers for forming the ATPS; for example, changing (a) DEX T40 (Mw~40,000) to (b) DEX T500 (Mw~500,000) and changing (a) PEG 8K (Mw~8,000) to (c) PEO (Mw~10,000).

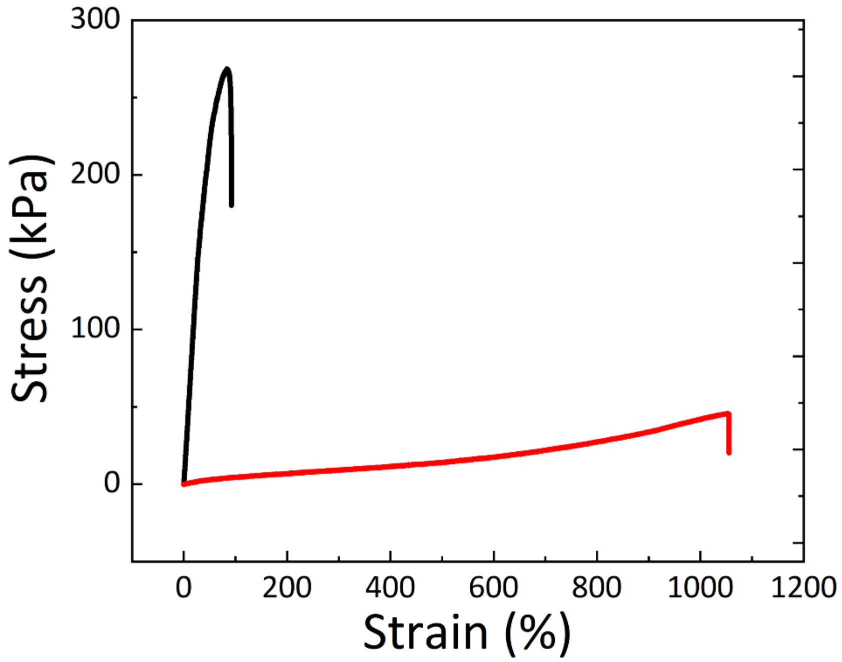

Supplementary Fig. 13: A representative stress-strain curve of TAP gel 2 hydrogel. The load is applied at a constant 50%/min strain rate while the curve is measured automatically by the software.

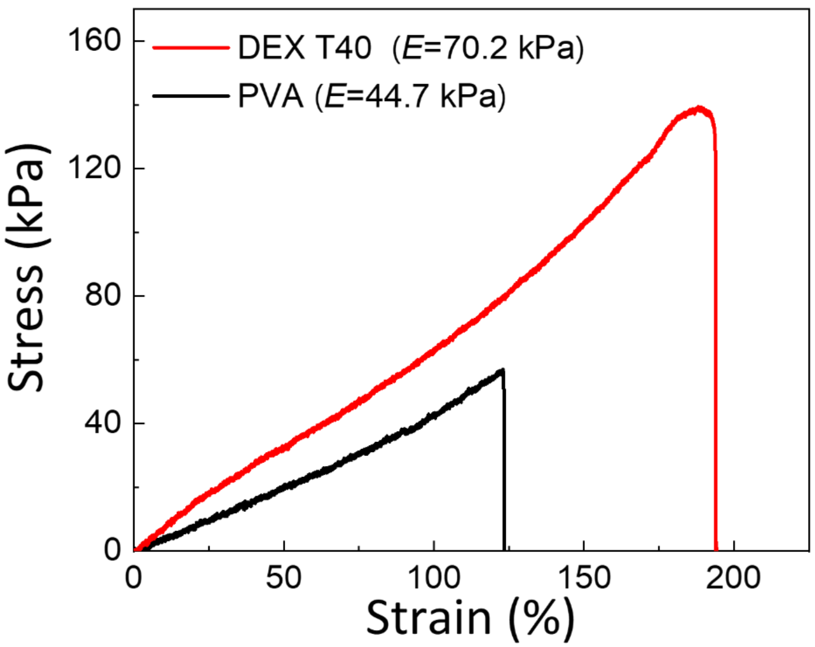

Supplementary Fig. 14: A representative stress-stain curve for TAP gel 3 without salting-out process comprised of 10 wt% PVA (Mw: 89,000 – 98,000, 99% hydrolyzed), 10 wt% Dextran T10, and 10 wt% acrylamide, which is further crosslinked with bis-acrylamide, at 1 wt% of the weight of acrylamide and Irgacure 2959, at 0.5 wt% of the weight of acrylamide. The red curve is DEX gel with $E_{DEX} = 70.2\ kPa$ and the black curve is PVA gel with $E_{PVA} = 44.\ 7\ kPa$.

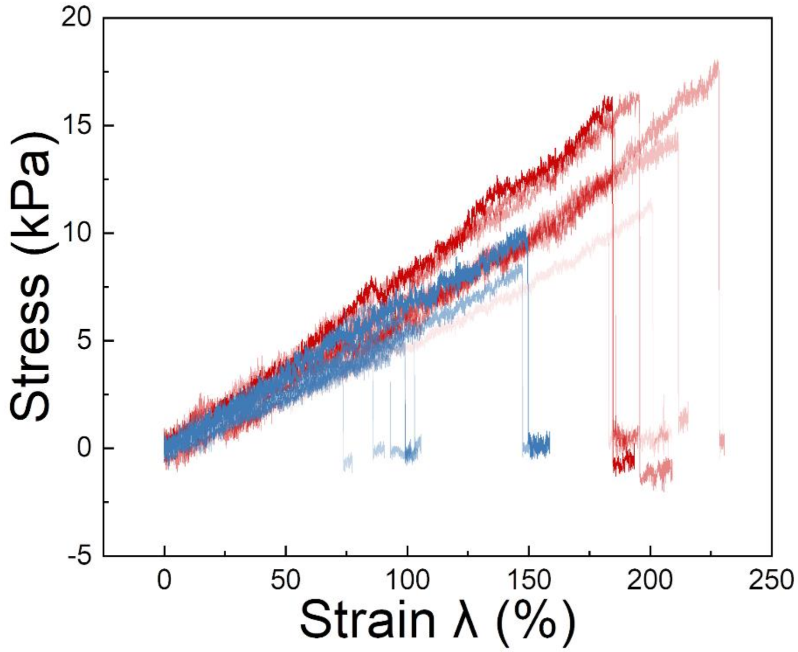

Supplementary Fig. 15: Stress-strain curve of ATPS-1 hydrogel fabricated by the conventional method (blue curves) and our method (red curves). Six samples are measured respectively for each method to get an average values and minimize errors.

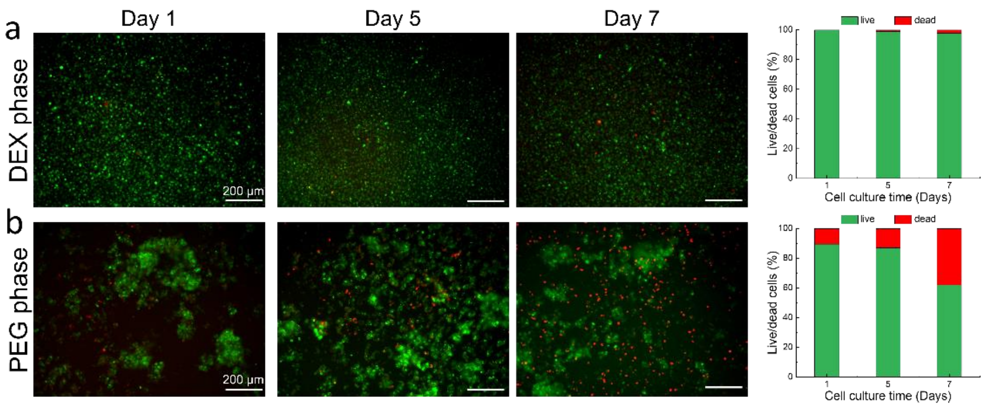

Supplementary Fig. 16: Long-term survival of HUVEC cells cultured in PEG phase and DEX phase of TAP gels, respectively. Fluorescence images show live (green) and dead (red) cells on day 1, day3, and day 7. Quantified results of cell viability show cells can survive in TAP gels up to 7 days.

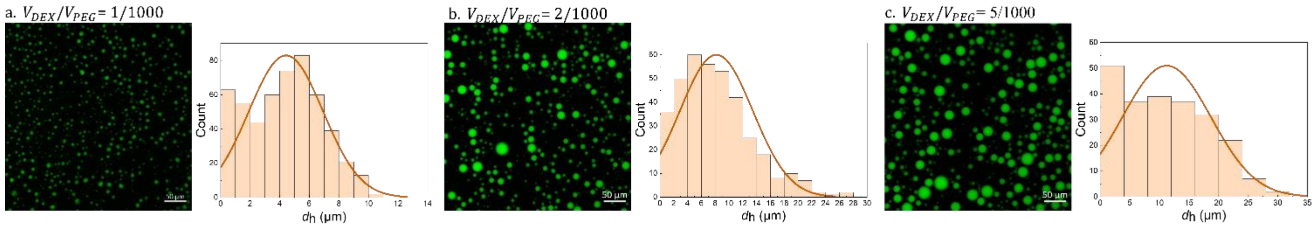

Supplementary Fig. 17: Fluorescent images and size distribution histograms of DEX-rich droplets dispersed within PEG-rich phase at different respective volume ratios.

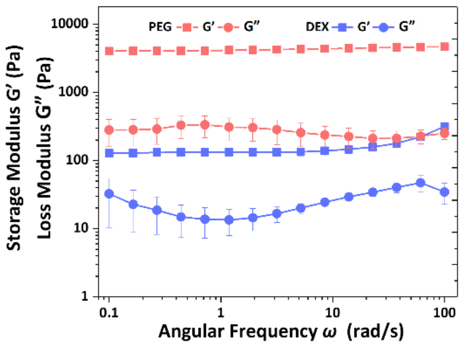

Supplementary Fig. 18: Storage (G') and loss (G'') moduli of the PEG-rich gel and DEX-rich gel. Samples were measured with a constant strain γ=1 % in their linear viscoelastic regime. Error bras indicate mean ±SD (n=3 independent samples).

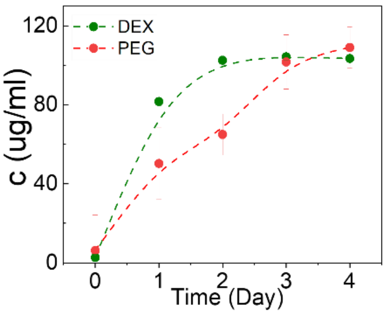

Supplementary Fig. 19. Release profiles of FITC-labelled DEX from DEX-rich phase and Rhodamine-labelled PEG from PEG-rich phase. Error bras indicate mean ±SD (n=3 independent samples).

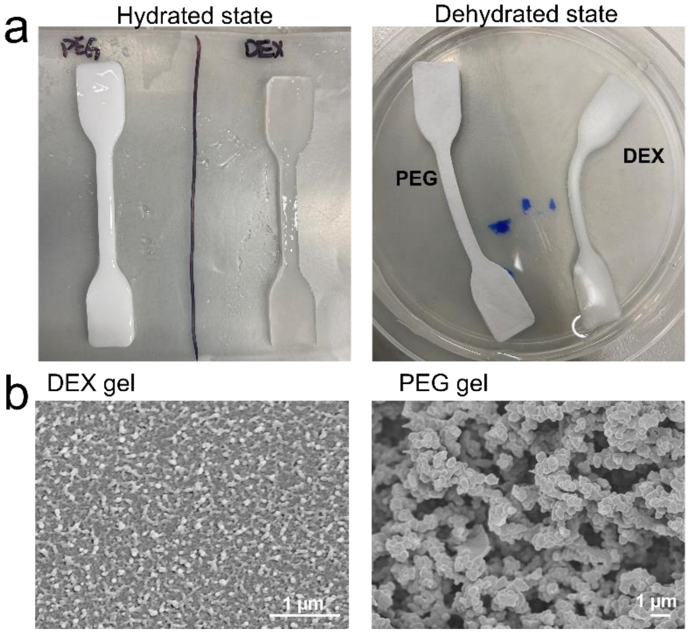

Supplementary Fig. 20: (a) Photos of PEG and DEX hydrogel at hydrated state and dehydration state. Hydrogel samples were completely dehydrated by replacing water with absolute ethanol for more than 2 days. Absolute ethanol was frequently exchanged every 12 hours. (b) SEM images of pore structures in the (a) DEX gel and (b) PEG gel showing no clear pore structures.

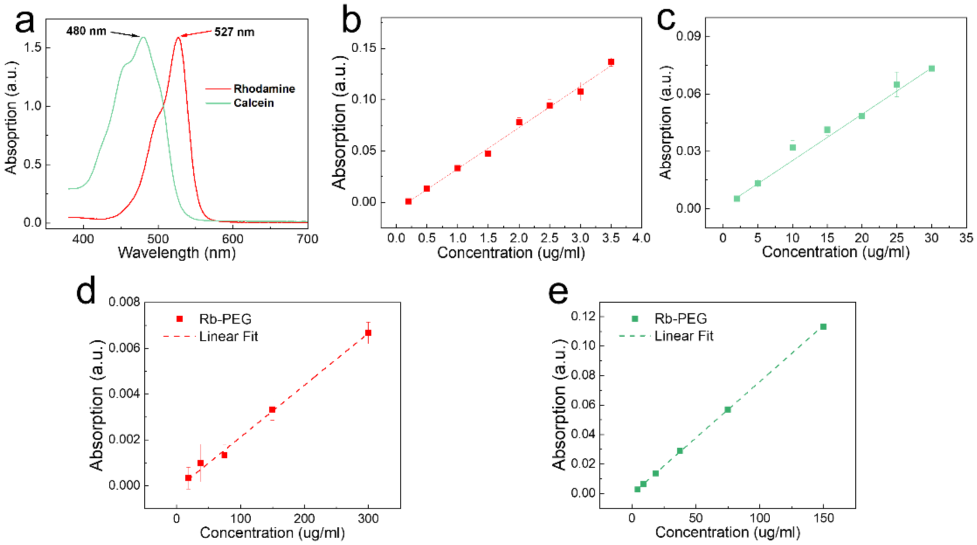

Supplementary Fig. 21: (a) Absorption spectrum of Rhodamine and calcein to identify their highest absorbing wavelength at 527 nm and 480 nm, respectively. Calibration curves of (b) rhodamine and (c) calcein are plotted at their highest absorbing wavelengths. Calibration curves of (d) Rb-PEG and (e) FITC-DEX are plotted as absorption versus concentration measured absorbing wavelength of 590 nm and 450 nm, respectively. Error bras indicate mean ±SD (n=3 independent samples). The unit of a.u. represents absorption unit acquired from the instrument.

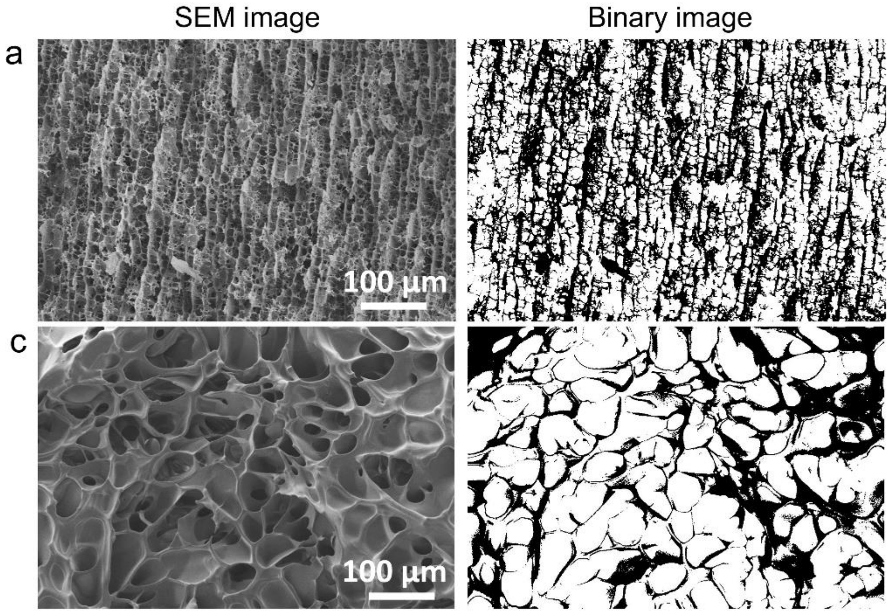

Supplementary Fig. 22: SEM and binary images of (a) DEX and (b) PEG. The white area represents porous structures and black areas represent polymer networks. The porosity is calculated as the ratio of while area to black area.

Supplementary References

1. H.-O. Johansson, E. Feitosa, A. P. J. P. Junior, Phase diagrams of the aqueous two-phase systems of poly (ethylene glycol)/sodium polyacrylate/salts. *Polymer* **3**, 587-601 (2011).
2. B. C. Bussamra, D. Sietaram, P. Verheijen, S. I. Mussatto, A. C. da Costa, L. van der Wielen, M. Ottens, A critical assessment of the Flory-Huggins (FH) theory to predict aqueous two-phase behaviour. *Sep. Purif. Technol.* **255**, 117636 (2021).
3. M. Iqbal, Y. Tao, S. Xie, Y. Zhu, D. Chen, X. Wang, L. Huang, D. Peng, A. Sattar, M. A. B. Shabbir, Aqueous two-phase system (ATPS): an overview and advances in its applications. *Biol. Proced. Online* **18**, 1-18 (2016).
