# Supplementary material for: Phase-separation facilitated one-step fabrication of multiscale heterogeneous two-aqueous-phase gel
Source: Nat Commun. 2023 May 16;14:2793. doi: 10.1038/s41467-023-38394-9 (PMC10188440; doi:10.1038/s41467-023-38394-9)
Supplement: Supplementary file 2 — Reporting Summary [file 41467_2023_38394_MOESM2_ESM.pdf]

nature portfolio

Corresponding author(s): Ho Cheung Shum

Last updated by author(s): Apr 12, 2023

Reporting Summary

Nature Portfolio wishes to improve the reproducibility of the work that we publish. This form provides structure for consistency and transparency in reporting. For further information on Nature Portfolio policies, see our Editorial Policies and the Editorial Policy Checklist.

Statistics

For all statistical analyses, confirm that the following items are present in the figure legend, table legend, main text, or Methods section.

n/a	Confirmed	
☐	☒	The exact sample size (n) for each experimental group/condition, given as a discrete number and unit of measurement
☐	☒	A statement on whether measurements were taken from distinct samples or whether the same sample was measured repeatedly
☒	☐	The statistical test(s) used AND whether they are one- or two-sided<br>*Only common tests should be described solely by name; describe more complex techniques in the Methods section.*
☒	☐	A description of all covariates tested
☒	☐	A description of any assumptions or corrections, such as tests of normality and adjustment for multiple comparisons
☐	☒	A full description of the statistical parameters including central tendency (e.g. means) or other basic estimates (e.g. regression coefficient) AND variation (e.g. standard deviation) or associated estimates of uncertainty (e.g. confidence intervals)
☒	☐	For null hypothesis testing, the test statistic (e.g. F, t, r) with confidence intervals, effect sizes, degrees of freedom and P value noted<br>*Give P values as exact values whenever suitable.*
☒	☐	For Bayesian analysis, information on the choice of priors and Markov chain Monte Carlo settings
☒	☐	For hierarchical and complex designs, identification of the appropriate level for tests and full reporting of outcomes
☒	☐	Estimates of effect sizes (e.g. Cohen's d, Pearson's r), indicating how they were calculated

Our web collection on statistics for biologists contains articles on many of the points above.

Software and code

Policy information about availability of computer code

Data collection	Optical, fluorescence and 3D images were captured by a confocal laser scanning microscope (Carl Zesis LSM 700).<br>SEM images of hydrogels were captured by Hitachi S4800 FEG.<br>ZwickRoell software was used to acquire data for the mechanical testament of hydrogels.<br>Size distribution of droplets was measured through dynamic light scattering (DLS) using Zetasizer Pro (Malvern Instruments, UK)<br>Absorption intensity of solutions over time was measured by a microplate reader (Molecular Devices)
Data analysis	Image binarization was performed by Fiji Image J (https://imagej.net/software/fiji/downloads).<br>Microscopic image data analysis was performed by ZEN software.<br>All data plots and histograms were performed by Origin 2021.

For manuscripts utilizing custom algorithms or software that are central to the research but not yet described in published literature, software must be made available to editors and reviewers. We strongly encourage code deposition in a community repository (e.g. GitHub). See the Nature Portfolio guidelines for submitting code & software for further information.

Data

Policy information about availability of data

All manuscripts must include a data availability statement. This statement should provide the following information, where applicable:

- Accession codes, unique identifiers, or web links for publicly available datasets
- A description of any restrictions on data availability
- For clinical datasets or third party data, please ensure that the statement adheres to our policy

All data needed to evaluate the conclusions in the paper are present in the paper and/or the Supplementary Information, and also from the corresponding author upon request. All data generated in this study are provided in Source Data file.

Human research participants

Policy information about studies involving human research participants and Sex and Gender in Research.

Reporting on sex and gender	Not applicable in this study.
Population characteristics	Not applicable in this study.
Recruitment	Not applicable in this study.
Ethics oversight	Not applicable in this study.

Note that full information on the approval of the study protocol must also be provided in the manuscript.

Field-specific reporting

Please select the one below that is the best fit for your research. If you are not sure, read the appropriate sections before making your selection.

☒ Life sciences ☐ Behavioural & social sciences ☐ Ecological, evolutionary & environmental sciences

For a reference copy of the document with all sections, see nature.com/documents/nr-reporting-summary-flat.pdf

Life sciences study design

All studies must disclose on these points even when the disclosure is negative.

Sample size	No statistical method was used to predetermine the sample size. For each experiment, we typically performed at least three samples to calculate average value and standard derivation. When observing small variations in the data, three independent samples were sufficient to confirm reproducibility. However, for large variations in the data, we performed additional independent tests to ensure reproducibility and get mean values and statistical analysis.
Data exclusions	No data were excluded from the analysis.
Replication	All experiments were reliably reproduced and all attempts at replication were successful. Experiments were repeated to be successful over a course of approximately 2 years during the development of this work. Detailed fabrication methods were provided to aid in replications by readers.
Randomization	Randomization was not relevant to this study as there were no confounding variables that need to be controlled.
Blinding	Investigators were blinded to group allocation during data collection and data analysis as the analysis, which was performed in an automated way by software.

Reporting for specific materials, systems and methods

We require information from authors about some types of materials, experimental systems and methods used in many studies. Here, indicate whether each material, system or method listed is relevant to your study. If you are not sure if a list item applies to your research, read the appropriate section before selecting a response.

Materials & experimental systems

n/a	Involved in the study
☒	☐ Antibodies
☐	☒ Eukaryotic cell lines
☒	☐ Palaeontology and archaeology
☒	☐ Animals and other organisms
☒	☐ Clinical data
☒	☐ Dual use research of concern

Methods

n/a	Involved in the study
☒	☐ ChIP-seq
☒	☐ Flow cytometry
☒	☐ MRI-based neuroimaging

Eukaryotic cell lines

Policy information about cell lines and Sex and Gender in Research

Cell line source(s)	3T3 cells (NIH-3T3 mouse embryonic fibroblast cells, Beyotime, Cat# C7204), HUVEC cells (Human Umbilical Vein Endothelial Cells, Thermo Fisher Scientific, Cat# C0035C) were used.
Authentication	None of the cell lines used were authenticated
Mycoplasma contamination	All cell lines were confirmed without mycoplasma contamination.
Commonly misidentified lines (See ICLAC register)	None
